# New insight into the mechanisms of ectopic fat deposition improvement after bariatric surgery

**DOI:** 10.1038/s41598-019-53702-4

**Published:** 2019-11-21

**Authors:** Giulia Angelini, Lidia Castagneto Gissey, Giulia Del Corpo, Carla Giordano, Bruna Cerbelli, Anna Severino, Melania Manco, Nicola Basso, Andreas L. Birkenfeld, Stefan R. Bornstein, Alfredo Genco, Geltrude Mingrone, Giovanni Casella

**Affiliations:** 1 Fondazione Policlinico Universitario A. Gemelli IRCCS, Rome, Italy and Università Cattolica del S. Cuore, Rome, Italy; 2 Department of Surgical Sciences, Sapienza University of Rome, Rome, Italy; 3 Department of Radiological, Oncological and Pathological Sciences, Sapienza University of Rome, Rome, Italy; 4 Research Unit for Multi-factorial Diseases, Obesity and Diabetes, Istituti di Ricovero e Cura a Carattere Scientifico, Bambino Gesù Children’s Hospital, Rome, Italy; 5 Department of Medicine III, Universitätsklinikum Carl Gustav Carus an der Technischen Universität Dresden, Dresden, Germany; 6 Paul Langerhans Institute Dresden of the Helmholtz Center Munich at University Hospital and Faculty of Medicine, TU Dresden, Dresden, Germany; 7 Diabetes and Nutritional Sciences, King’s College London, London, United Kingdom; 8 Deutsches Zentrum für Diabetesforschung, DZD e.V., Neuherberg, Germany

**Keywords:** Molecular biology, Obesity

## Abstract

Non-alcoholic fatty-liver disease (NAFLD) is frequent in obese patients and represents a major risk factor for the development of diabetes and its complications. Bariatric surgery reverses the hepatic features of NAFLD. However, its mechanism of action remains elusive. We performed a comprehensive analysis of the mechanism leading to the improvement of NAFLD and insulin resistance in both obese rodents and humans following sleeve-gastrectomy (SG). SG improved insulin sensitivity and reduced hepatic and monocyte fat accumulation. Importantly, fat accumulation in monocytes was well comparable to that in hepatocytes, suggesting that Plin2 levels in monocytes might be a non-invasive marker for the diagnosis of NAFLD. Both *in vitro* and *in vivo* studies demonstrated an effective metabolic regeneration of liver function and insulin sensitivity. Specifically, SG improved NAFLD significantly by enhancing AMP-activated protein kinase (AMPK) phosphorylation and chaperone-mediated autophagy (CMA) that translate into the removal of Plin2 coating lipid droplets. This led to an increase in lipolysis and specific amelioration of hepatic insulin resistance. Elucidating the mechanism of impaired liver metabolism in obese subjects will help to design new strategies for the prevention and treatment of NAFLD.

## Introduction

Non-alcoholic fatty liver disease (NAFLD) affects about 25% of the general population with peaks of 32% in Middle East and 30% in South America^1^. A recent study demonstrated a linear relationship between body mass index (BMI) and NAFLD diagnosis, which was about 5- to 9-fold higher in subjects with a BMI between 30 and 32.5 kg/m^2^ increasing to ca. 10- to 14-fold at BMIs of 37.5–40 kg/m^2^ as compared with normal weight subjects^2^. The prevalence of NAFLD in type 2 diabetes (T2D) is estimated to be around 60%^3^.

Non-alcoholic steatohepatitis (NASH) is a more aggressive form of NAFLD characterized by the presence of inflammatory cell infiltration and hepatocyte ballooning, which may further progress to cirrhosis and/or to hepatocellular carcinoma^1^.

Currently, there are no drugs approved to specifically treat NAFLD or NASH and thus weight loss remains the only approach.

Bariatric surgery (BS) promotes T2D remission both in the short and in the long term^4–10^, as well as remission of both NAFLD and NASH^11^.

The major effect of SG is to reduce energy intake. It is known that a reduced energy intake activates AMP-activated protein kinase (AMPK) in its phosphorylated form that tends to counterbalance a low energy status and restores energy balance through the inhibition of ATP consumption and simultaneous promotion of ATP synthesis^12,13^. Ezquerro *et al*.^14^ found that in rats under a high-fat diet sleeve gastrectomy improved NAFLD by reducing hepatosteatosis and circulating transaminase levels through the downregulation of lipogenesis and the increase of both autophagy and mitochondrial β-oxidation.

NAFLD is characterized by the hepatocyte accumulation of neutral lipids forming lipid droplets (LDs), known as hepatic steatosis, surrounded by proteins of which Perilipin 2 (Plin2) is a major component^15^. Plin2 deletion or suppression of Plin2 expression protect from NAFLD development in genetic models of obesity or in rodents fed a high-fat diet (HFD)^16,17^.

Chaperone-mediated autophagy (CMA) promotes Plin2 catabolism and, therefore, contributes to LD degradation by facilitating lipolysis^18^. The heat shock cognate protein of 70 kDa (Hsc70) recognizes the pentapeptide motif of Plin2^19^ and transports this protein to the lysosome where Plin2 binds to the CMA receptor, LAMP2A (lysosome-associated membrane protein 2A), and is translocated into the lysosomal lumen where is degraded^20^. An important step that allows the binding of Plin2 to LAMP2A is its previous phosphorylation that is dependent on AMPK.

We hypothesize that the energy imbalance generated by SG upregulates liver AMPK and promotes Plin2 degradation leading to the improvement of NAFLD/NASH.

Therefore, we explored these mechanisms performing SG in diet-induced obesity, NAFLD in rats and in subjects with NAFLD.

We observed that ectopic fat deposition in rodents with diet-induced obesity (DIO) included not only hepatic steatosis but also lipid deposition in circulating monocytes in concert with increased Plin2 expression. We found that SG increased AMPK Thr172 phosphorylation, LAMP2A expression and reduced Plin2 expression in both hepatocytes and monocytes. SG reduced the number of lipid droplets and increased the glycogen storage both in hepatocytes and monocytes; Akt Ser473, Glycogen Synthase Kinase (GSK3αβ) Ser21/9 and Forkhead Box O1 (FoxO1) Thr24 phosphorylation were all increased. Plin2 overexpression in primary cultures of hepatocytes and monocytes reduced Akt phosphorylation and lead to insulin resistance.

Together, these data suggest that liver fat accumulation and hepatic insulin resistance share a common pathway coordinated by Plin2.

## Results

### Animal study

#### SG reduces hepatic fat accumulation in diet-induced obesity and significantly reduced Plin2 protein expression in both liver and monocytes

**Figure 1:**
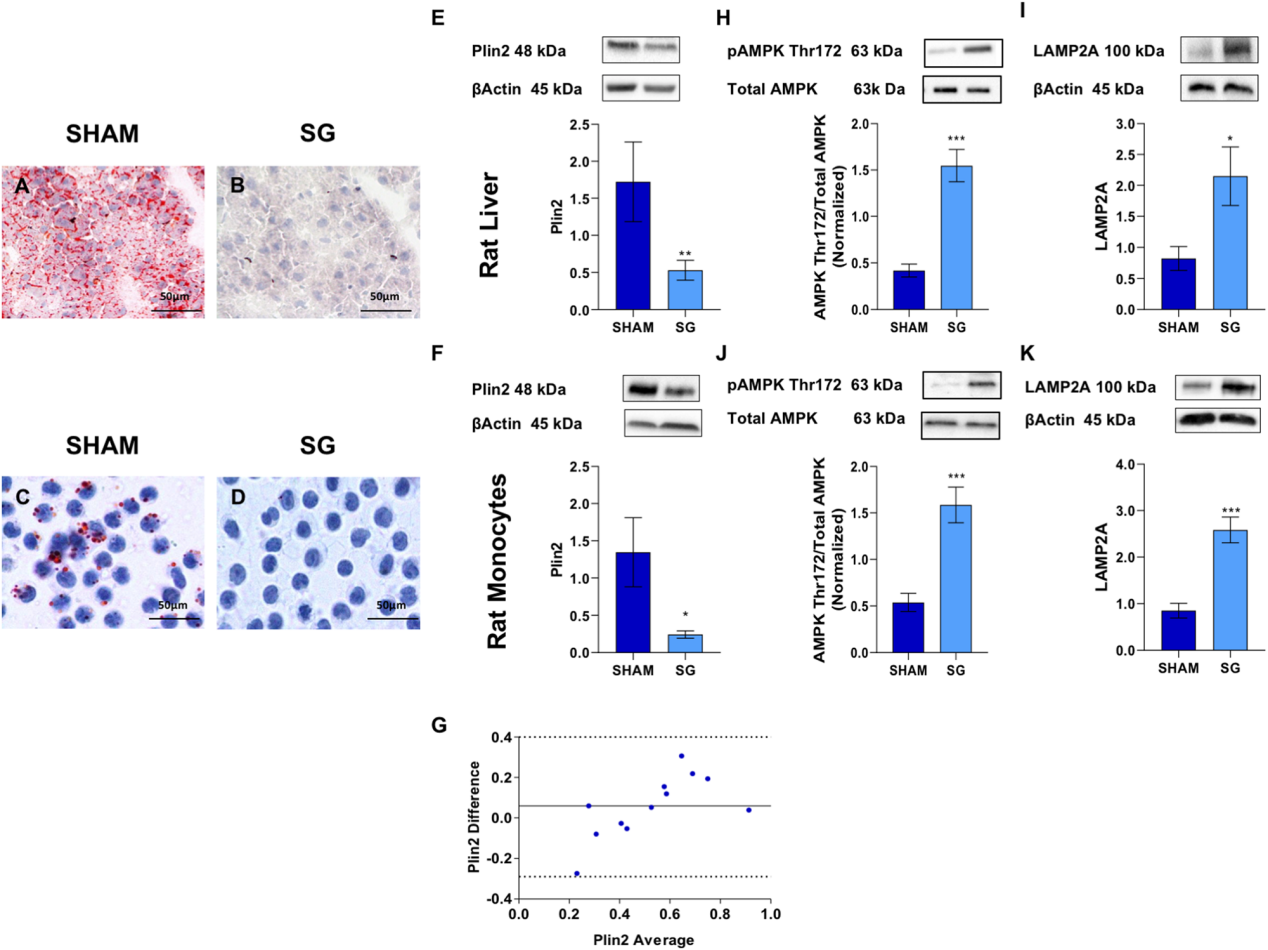
SG reduces hepatic fat accumulation in diet-induced obesity rats and significantly reduced Plin2 protein expression in both liver and monocytes. Panels (A–D): Oil Red O staining of liver biopsies (**C**,**D**) and monocytes (**E**,**F**) from sham and SG rats; SG drastically reduced hepatic fat accumulation. Panels (E,F): Western blot analysis of Plin2 protein level, in liver biopsies and monocytes of sham and SG rats. SG significantly reduced Plin2 levels in both liver and monocytes of DIO rats (Liver: 1.70 ± 0.50 vs. 0.53 ± 0.10, P = 0.008; Monocytes:1.34 ± 0.50 vs. 0.24 ± 0.05, P = 0.01). Panel (G): Bland-Altman plot shows a good agreement between the two measures of Plin2 in the liver and in monocytes. Panels (H–K): SG significantly increased AMPK Thr172 phosphorylation and LAMP2A in both liver (**H**,**I**) (AMPK Thr172: 0.42 ± 0.0.07 vs. 1.55 ± 0.17, P = 0.0001; LAMP2A: 0.82 ± 0.19 vs. 2.15 ± 0.47, P = 0.02) and monocytes (**J**,**K**) (AMPK Thr172: 0.54 ± 0.01 vs. 1.59 ± 0.19, P = 0.0001; LAMP2A: 0.85 ± 0.16 vs. 2.60 ± 0.30, P = 0.0003). Data are expressed as mean ± SEM (n = 15 rat per group).

Body weight was significantly lower in the SG than in the sham group (534.10 ± 25.71 vs. 336.40 ± 10.31 g, P = 0.0003). Compared with sham operation, SG rats drastically reduced fat accumulation in both liver and monocytes of DIO rats (Fig. 1, Panels A–D).

SG significantly reduced Plin2 levels in both liver and monocytes of DIO rats (Liver: 1.70 ± 0.50 vs. 0.53 ± 0.10, P = 0.008; Monocytes:1.34 ± 0.50 vs. 0.24 ± 0.05, P = 0.01) (Fig. 1, Panels E,F). The Bland-Altman plot in Fig. 1, Panel G, showed a good agreement between the measures of Plin2 in the liver and in monocytes. AMPK Thr172 phosphorylation and LAMP2A levels were higher in both liver (AMPK Thr172: 0.42 ± 0.0.07 vs. 1.55 ± 0.17, P = 0.0001; LAMP2A: 0.82 ± 0.19 vs. 2.15 ± 0.47, P = 0.02) (Fig. 1 Panel H,I) and monocytes (AMPK Thr172: 0.54 ± 0.01 vs. 1.59 ± 0.19, P = 0.0001; LAMP2A: 0.85 ± 0.16 vs. 2.60 ± 0.30, P = 0.0003) after SG as compared with sham operation (Fig. 1 Panel J,K).

#### SG increased insulin sensitivity influencing Akt, GSK3αβ and FoxO1 phosphorylation

**Figure 2:**
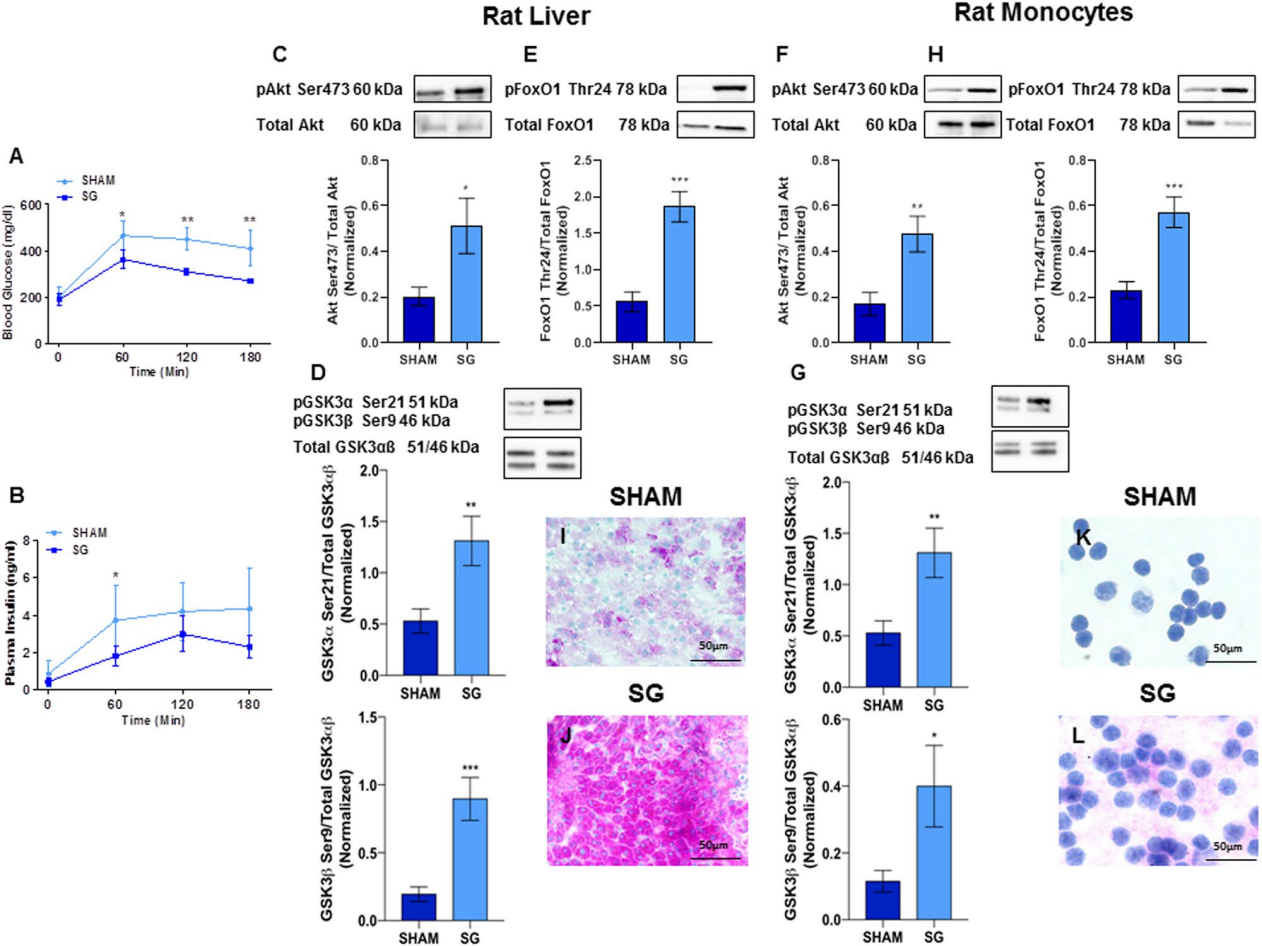
SG significantly improves insulin signaling. Panels (A,B): Time courses of blood glucose (**A**) and plasma insulin (**B**) in sham and SG rats. SG significantly reduced both blood glucose and plasma insulin concentrations. *P < 0.04; **P = 0.004. Panels (C–E): Liver Akt Ser473, GSK3αβ Ser21/9 and FoxO1 Thr24 phosphorylation was significantly higher in DIO rats than in SG rats (Akt Ser473: 0.20 ± 0.04 vs. 0.51 ± 0.12, P = 0.02; GSK3α Ser21: 0.53 ± 0.12 vs. 1.31 ± 0.24, P = 0.006; GSK3β Ser9: 0.19 ± 0.05 vs. 0.90 ± 0.16, P = 0.0003: FoxO1 Thr24: 0.56 ± 0.13 vs. 1.86 ± 0.20, P = 0.0001). Panels (F–H): SG increased monocytes Akt Ser473, GSK3αβ Ser21/9 and FoxO1 Thr24 phosphorylation in DIO rats (Akt Ser473: 0.17 ± 0.05 vs. 0.47 ± 0.08, P = 0.004; GSK3α Ser21: 0.53 ± 0.12 vs. 1.31 ± 0.24, P = 0.006; GSK3β Ser9: 0.11 ± 0.03 vs. 0.40 ± 0.12, P = 0.03: FoxO1 Thr24: 0.23 ± 0.04 vs. 0.57 ± 0.07, P = 0.0006). Panels (I–L): Periodic acid-Schiff staining of liver biopsies (**I**,**J**) and monocytes (**K**,**L**) from sham and SG rats; SG markedly increased hepatic glycogen depots. Data are expressed as mean ± SEM (n = 15 rats per group).

The time courses of blood glucose and plasma insulin after the OGTT are shown in Fig. 2, Panels A,B, respectively. Both blood glucose (73342.50 ± 8671.01 vs. 46627.50 ± 4487.65 mg/dl *x* min, P = 0.007) and plasma insulin AUCs (611.31 ± 186.74 vs. 371.64 ± 73.31 ng/ml *x* min, P = 0.017) were almost halved in the SG group as compared with the sham group. Furthermore, we found that Akt Ser473, GSK3αβ Ser21/9 and FoxO1 Thr24 phosphorylation were significantly higher in both liver (Akt Ser473: 0.20 ± 0.04 vs. 0.51 ± 0.12, P = 0.02; GSK3α Ser21: 0.53 ± 0.12 vs. 1.31 ± 0.24, P = 0.006; GSK3β Ser9: 0.19 ± 0.05 vs. 0.90 ± 0.16, P = 0.0003: FoxO1 Thr24: 0.56 ± 0.13 vs. 1.86 ± 0.20, P = 0.0001) (Fig. 2, Panels C–E) and monocytes (Akt Ser473: 0.17 ± 0.05 vs. 0.47 ± 0.08, P = 0.004; GSK3α Ser21: 0.53 ± 0.12 vs. 1.31 ± 0.24, P = 0.006; GSK3β Ser9: 0.11 ± 0.03 vs. 0.40 ± 0.12, P = 0.03: FoxO1 Thr24: 0.23 ± 0.04 vs. 0.57 ± 0.07, P = 0.0006) of DIO rats undergone SG than in sham-operated rats Fig. 2, Panels F–H). Compared with sham operation, SG rats drastically increased glycogen depots in both liver and in monocytes (Fig. 2, Panels I–L).

### Human study

The anthropometric characteristics of the obese patients, before and after SG, as well as those of controls are summarized in Table 1. The weight reduction was ca. 32% but the weight of the patients remained significantly higher than that of controls. Sleeve gastrectomy improved significantly the lipid profile and reduced significantly transaminases.

**Table 1:** Clinical characteristics of the study population (mean ± SEM.).

	Before Metabolic Surgery	P value Before/After	After Metabolic Surgery	P value Before Surgery vs. Controls	Controls	P value After Surgery vs. Controls
Number of patients	15		15		8	
Age (years)	39.7 ± 2.1	NS	40.67 ± 2.1	NS	39.9 ± 2.9	NS
Weight (kg)	117.4 ± 4.8	< 0.0001	79.6 ± 2.1	< 0.0001	64.7 ± 3.2	0.0006
BMI (kg/m^2^)	43.0 ± 0.9	< 0.0001	29.2 ± 0.7	< 0.0001	21.5 ± 0.74	< 0.0001
Plasma Glucose (mg/dl)	94.1 ± 2.3	0.01	86.7 ± 1.4	< 0.0001	74.8 ± 2.1	< 0.0001
Plasma Insulin (mU/l)	16.0 ± 3.7	0.02	6.1 ± 1.3	NS	8.2 ± 0.62	NS
HOMA-IR	3.6 ± 0.8	0.02	1.3 ± 0.3	0.02	1.5 ± 0.11	NS
HDL-cholesterol (mg/dl)	40.0 ± 4.3	0.01	56.0 ± 4.4	0.0008	64.6 ± 4.8	NS
*LDL-cholesterol (mg/dl)	137.3 ± 8.6	0.0002	93.9 ± 5.7	0.0004	95.2 ± 4.0	NS
Triglycerides (mg/dl)	146.2 ± 15.6	0.0003	72.3 ± 7.7	0.03	105.7 ± 8.4	0.007
AST (IU/l)	57.5 ± 4.7	<0.0001	29.2 ± 1.7	< 0.0001	18.6 ± 1.8	NS
ALT (IU/l)	32.3 ± 5.4	0.02	18.5 ± 1.16	0.02	17.7 ± 0.63	NS
NAS score	6.07 ± 0.63					
Excess Weight (g)	49.57 ± 3.35	<0.0001	11.76 ± 1.79			
Excess Weight Loss (%)			75.63 ± 3.42			

^*^LDL-cholesterol (mg dL^−1^) = total cholesterol (mg dL^−1^) − HDL-cholesterol (mg dL^−1^) – triglycerides (mg dL^−1^)/5.

#### Plin2 protein expression in monocytes is significantly reduced after sleeve gastrectomy and strongly correlates with the NAS score

**Figure 3:**
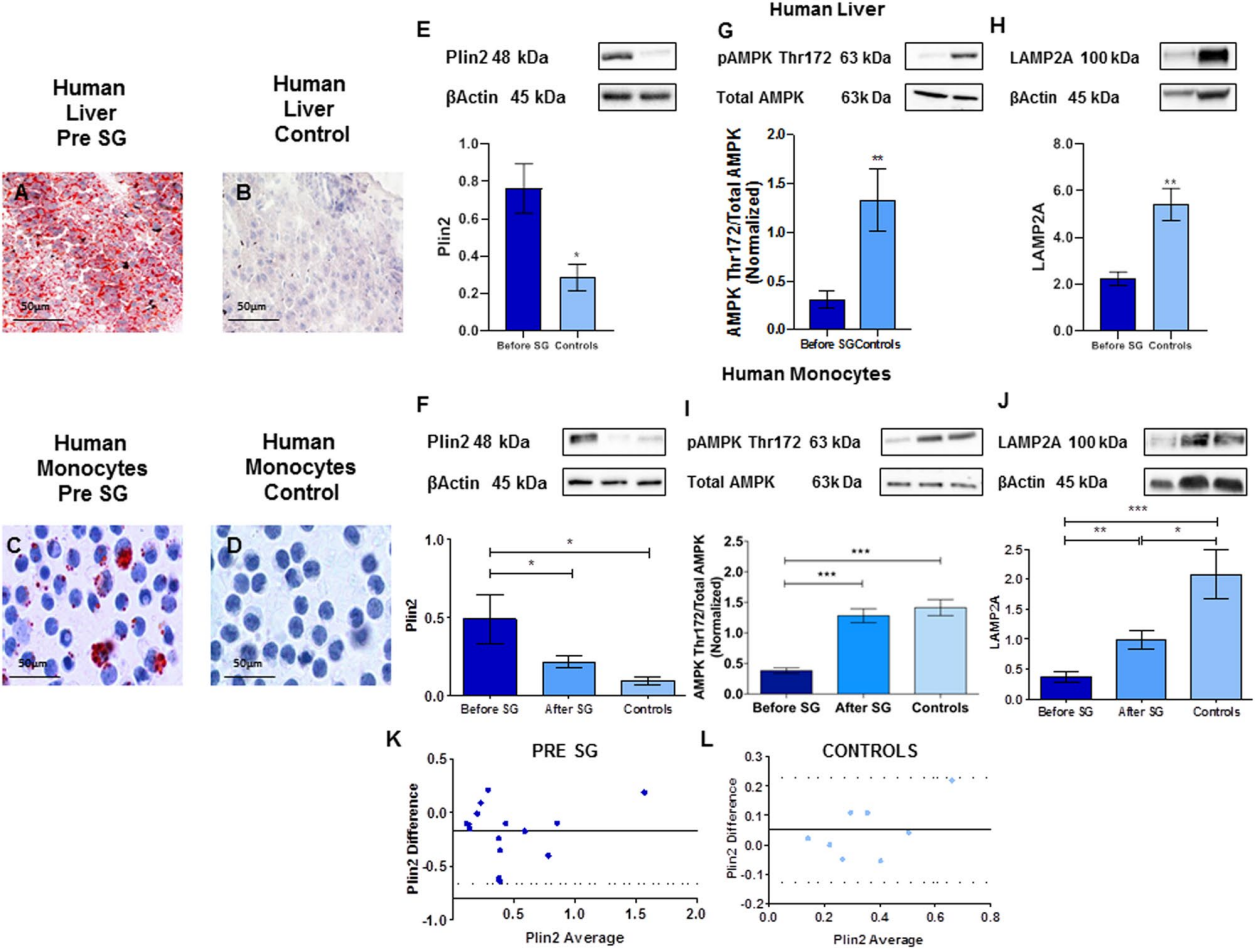
Plin2 protein expression in monocytes was significantly reduced after sleeve gastrectomy. Panels (A–D): Oil Red O staining of liver biopsies (**A**,**B**) and monocytes (**C**,**D**) of obese subjects with NAFLD before sleeve gastrectomy and controls. Fat accumulation was much higher in obese subjects than in controls. Panel (E): Plin2 protein expression in the liver was significantly lower in controls than in obese subjects with NAFLD (0.76 ± 0.13 vs. 0.28 ± 0.15, P = 0.03). Panel (F): Plin2 protein expression in monocytes decreased from 0.49 ± 0.16 vs. 0.21 ± 0.04, P = 0.01 after sleeve gastrectomy. Panels (G,H): AMPK Thr172 phosphorylation and LAMP2A expression in the liver was significantly lower in obese subjects with NAFLD than in controls (AMPK Thr172: 0.31 ± 0.09 vs. 1.33 ± 0.32, P = 0.006; LAMP2A: 2.23 ± 0.30 vs. 5.42 ± 0.70, P = 0.003). Panels (I,J): AMPK Thr172 phosphorylation and LAMP2A protein expression in monocytes were significantly lower before than after SG (AMPK Thr172: 0.38 ± 0.04 vs. 1.28 ± 0.11, P = 0.0001; LAMP2A: from 0.37 ± 0.08 to 1.00 ± 0.16, P = 0.003). Panels (K,L): Bland-Altman plots show a good agreement between the measures of Plin2 in liver and in monocytes of obese subjects with NAFLD and controls. Data are expressed as mean ± SEM (n = 15 obese subjects with NAFLD before and after SG and n = 8 controls).

Compared with controls, subjects before SG show high fat accumulation (Fig. 3, Panels A–D). Plin2 protein expression of both liver and monocytes significantly and positively correlated with the NAS score (Liver: R = 0.52, P = 0.0025; Monocytes: R = 0.58, P = 0.00099). Moreover, the expression of Plin2 in monocytes strongly and positively correlated with Plin2 expression in the liver (Before SG: R = 0.70, P < 0.0001; Controls: R = 0.90, P = 0.0007).

Figure 3, Panel E shows that Plin2 protein expression in the liver was significantly lower in controls than in obese subjects before SG (0.76 ± 0.13 vs. 0.28 ± 0.15, P = 0.03). Moreover, Plin2 protein expression in monocytes decreased from 0.49 ± 0.16 vs. 0.21 ± 0.04, P = 0.01) after SG (Fig. 3, Panel F).

AMPK Thr172 phosphorylation and LAMP2A expression in the liver was significantly lower in obese subjects than in controls (AMPK Thr172: 0.31 ± 0.09 vs. 1.33 ± 0.32, P = 0.006; LAMP2A: 2.23 ± 0.30 vs. 5.42 ± 0.70, P = 0.003) (Fig. 3, Panels G,H). Moreover, as shown in Fig. 3, Panels I,J, AMPK Thr172 phosphorylation and LAMP2A protein expression in monocytes were significantly lower before than after SG (AMPK Thr172: 0.38 ± 0.04 vs. 1.28 ± 0.11, P = 0.0001; LAMP2A: from 0.37 ± 0.08 to 1.00 ± 0.16, P = 0.003). The Bland-Altman plot in Fig. 3, Panels K,L, shows a good agreement between the measures of Plin2 in the liver and in monocytes of both obese subjects before sleeve gastrectomy and controls. Correlations of different variables with Plin2 are reported in Table 1 of Supplementary Materials.

#### Akt, GSK3αβ and FoxO1 phosphorylation is significantly higher in the liver of controls and increases after sleeve gastrectomy in monocytes

**Figure 4:**
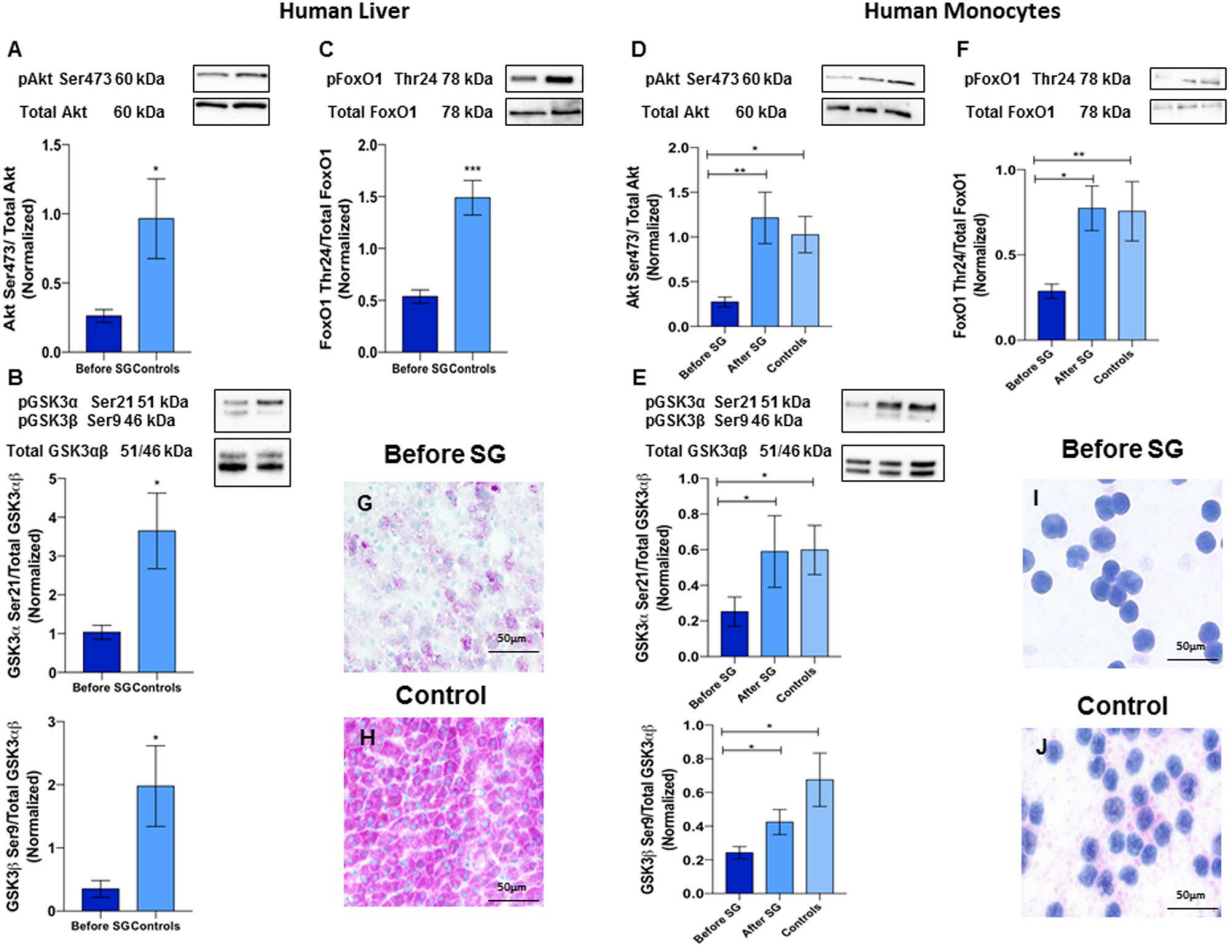
SG significantly affects insulin signaling. Panels (A–C): Liver Akt Ser473, GSK3αβ Ser21/9, FoxO1 Thr24 phosphorylation was significantly higher in controls than in obese subjects with NAFLD (Akt Ser473: 0.26 ± 0.04 vs 0.96 ± 0.29, P = 0.01; GSK3α Ser21: 1.03 ± 0.18 vs. 3.64 ± 0.97, P = 0.02; GSK3β Ser9: 0.35 ± 0.13 vs. 1.98 ± 0.63, P = 0.02; FoxO1 Thr24: 0.53 ± 0.06 vs. 1.49 ± 017, P = 0.001)). Panels (D–F): Akt Ser473, GSK3αβ Ser21/9 and FoxO1 Thr24 phosphorylation in monocytes is increased after SG (Akt Ser473: 0.27 ± 0.05 vs. 1.21 ± 0.29, P = 0.005; GSK3α Ser21: 0.25 ± 0.08 vs. 0.59 ± 0.20, P = 0.03; GSK3β Ser9: 0.24 ± 0.04 vs. 0.42 ± 0.07, P = 0.01; FoxO1 Thr24: 0.29 ± 0.04 vs. 0.77 ± 0.13, P = 0.001). Panels (G–J): Periodic acid-Schiff staining of liver biopsies (**G**,**H**) and monocytes (**I**,**J**) of obese subjects with NAFLD before sleeve gastrectomy and controls. Glycogen stores were much higher in controls than in obese subjects. Data are expressed as mean ± SEM (n = 15 obese subjects with NAFLD before and after SG and n = 8 controls).

Liver Akt Ser473, GSK3αβ Ser21/9, FoxO1 Thr24 phosphorylation were significantly higher in controls than in obese subjects at baseline (Akt Ser473: 0.26 ± 0.04 vs 0.96 ± 0.29, P = 0.01; GSK3α Ser21: 1.03 ± 0.18 vs. 3.64 ± 0.97, P = 0.02; GSK3β Ser9: 0.35 ± 0.13 vs. 1.98 ± 0.63, P = 0.02; FoxO1 Thr24: 0.53 ± 0.06 vs. 1.49 ± 017, P = 0.0001) (Fig. 4, Panels A–C). Moreover, Akt Ser473, GSK3αβ Ser21/9 and FoxO1 Thr24 phosphorylation in monocytes were increased after SG (Akt Ser473: 0.27 ± 0.05 vs. 1.21 ± 0.29, P = 0.005; GSK3α Ser21: 0.25 ± 0.08 vs. 0.59 ± 0.20, P = 0.03; GSK3β Ser9: 0.24 ± 0.04 vs. 0.42 ± 0.07, P = 0.01; FoxO1 Thr24: 0.29 ± 0.04 vs. 0.77 ± 0.13, P = 0.01) (Fig. 4, Panels D–F). Compared with controls, subjects before SG show lower glycogen depots in both liver and monocytes (Fig. 4, Panels G–J).

### *In vitro* studies

#### Fat accumulation in primary cultures of hepatocytes and monocytes from healthy controls

**Figure 5:**
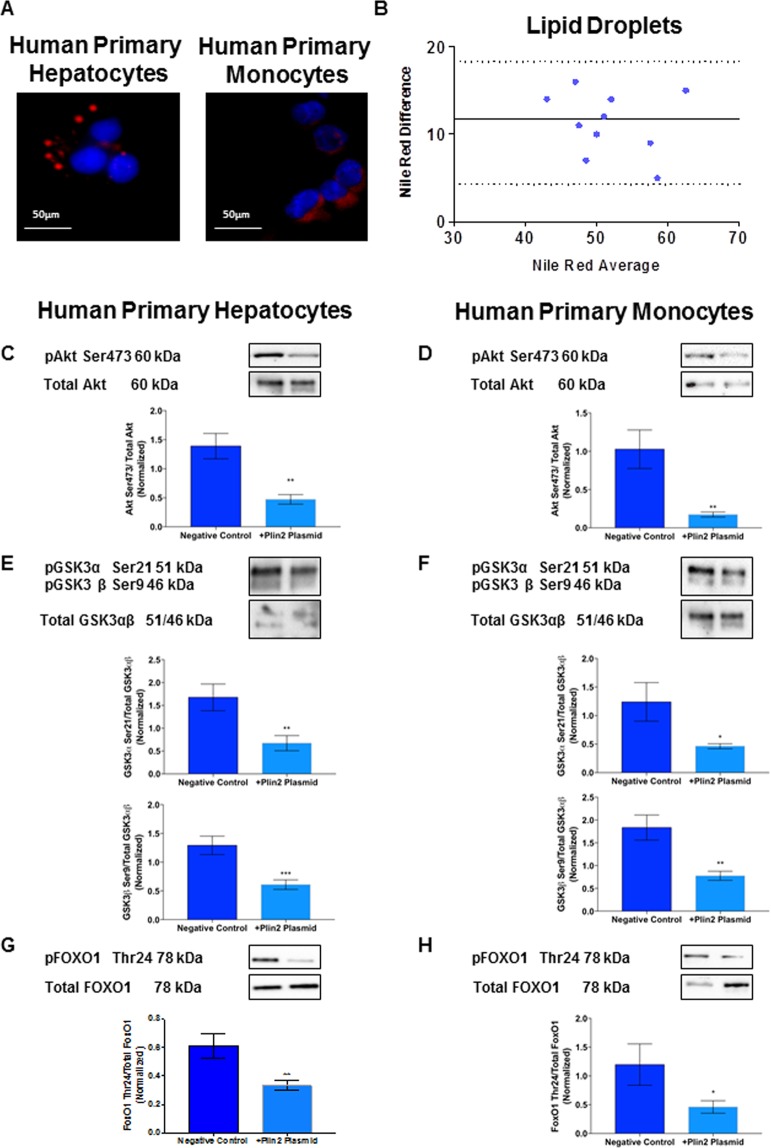
Overexpression of Plin2 decreases AKT Ser473, GSK3αβ Ser21/9 and FoxO1 Thr24 phosphorylation. Panel (A): Nile Red staining of liver biopsies and monocytes. Nile Red staining shows that ectopic fat accumulation occurs not only in hepatocytes but also in monocytes. Panel (B): Bland-Altman plot shows a good agreement between the measures of lipid droplets accumulation in hepatocytes and monocytes from healthy controls stimulated with oleic acid. Panels (C,D): Plin2 overexpression significantly reduced Akt Ser473 phosphorylation in hepatocytes (**C**), from 1.39 ± 0.22 to 0.47 ± 0.08, P = 0.002 and (**D**) in monocytes, from 1.03 ± 0.25 to 0.17 ± 0.03, P = 0.008. Panels (E,F): Plin2 overexpression significantly reduced GSK3α Ser21 phosphorylation in hepatocytes (**E**) from GSK3α Ser21: from 1.68 ± 0.29 to 0.67 ± 0.16, P = 0.001; GSK3β Ser9: from 1.29 ± 0.16 to 0.61 ± 0.08, P = 0.0001 and in monocytes (**F**), GSK3α Ser21: from 1.24 ± 0.34 to 0.46 ± 0.04, P = 0.04; GSK3β Ser9: from 1.85 ± 0.28 to 0.78 ± 0.01, P = 0.002. Panels (G,H): Plin2 overexpression significantly reduced FoxO1 Thr24 in hepatocytes (**G**) from 0 FoxO1 Thr24: from 0.61 ± 0.09 to 0.33 ± 0.04, P = 0.001 and in monocytes (**H**) from FoxO1 Thr24: from 1.20 ± 0.36 to 0.46 ± 0.11, P = 0.02. Data are expressed as mean ± SEM (n = 5 controls).

To confirm that ectopic fat accumulation occurs also in monocytes and to assess the amount of lipid droplets in primary hepatocytes and monocytes of healthy subjects, cells were exposed to the same concentration of oleic acid for 24 hours. Nile Red was used to stain hepatocytes and monocytes (Fig. 5, Panel A) and flow cytometry was used to quantify the amount of lipid droplet; the Bland-Altman plot showed a good agreement between the amount of lipid droplets in hepatocytes and monocytes (Fig. 5, Panel B).

#### AKT Ser473 GSK3αβ and FoxO1 phosphorylation significantly decrease by overexpressing Plin2 in primary cultures of hepatocytes and monocytes

In order to prove that a high Plin2 expression is responsible for the Akt Ser473, GSK3αβ Ser21/9 and FoxO1 Thr24 reduced phosphorylation, we overexpressed Plin2 by transfecting human Plin2 plasmid in primary cultures of hepatocytes and monocytes from controls.

As shown in Fig. 5, Panels C,E,G, Plin2 overexpression significantly reduced Akt Ser473, GSK3αβ Ser21/9 and FoxO1 Thr24 phosphorylation in primary cultures of hepatocytes (Akt Ser473: from 1.39 ± 0.22 to 0.47 ± 0.08, P = 0.002; GSK3α Ser21: from 1.68 ± 0.29 to 0.67 ± 0.16, P = 0.001; GSK3β Ser9: from 1.29 ± 0.16 to 0.61 ± 0.08, P = 0.0001; FoxO1 Thr24: from 0.61 ± 0.09 to 0.33 ± 0.04, P = 0.001. Additionally, Plin2 overexpression significantly reduced Akt Ser473, GSK3αβ Ser21/9 and FoxO1 Thr24 phosphorylation in monocytes of healthy controls (Akt Ser473: from 1.03 ± 0.25 to 0.17 ± 0.03, P = 0.008; GSK3α Ser21: from 1.24 ± 0.34 to 0.46 ± 0.04, P = 0.04; GSK3β Ser9: from 1.85 ± 0.28 to 0.78 ± 0.01, P = 0.002; FoxO1 Thr24: from 1.20 ± 0.36 to 0.46 ± 0.11, P = 0.02 (Fig. 5, Panels D,F,H). Supplementary Fig. 1, Panels A,C, shows the efficiency of cell transfection with pmaxGFP vector and Plin2 overexpression by flow cytometry (Supplementary Fig. 1, Panels B,D).

#### Correlations

We did not find significant correlations between changes in the phosphorylation of protein of interest, namely, AMPK Thr172, LAMP2A, Akt Ser473, GSK3αβ Ser21/9 and FoxO1 Thr24 and changes in body weight in both humans and rodents.

#### Liver insulin sensitivity improvement induced by metformin or pioglitazone reduces Plin2 levels in primary hepatocyte cultures via CMA activation

**Figure 6:**
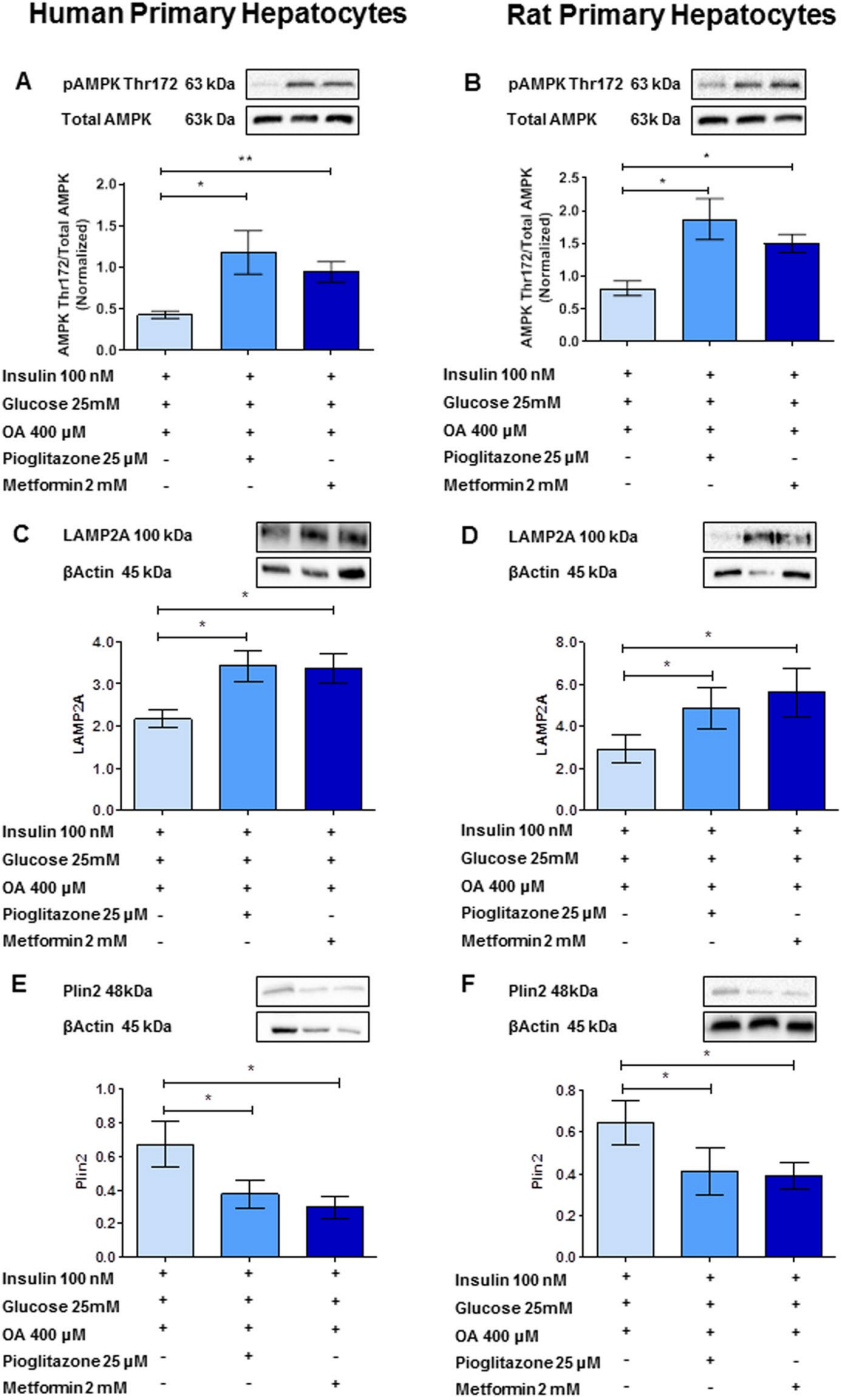
Pioglitazone or metformin reduced Plin2 levels in primary hepatocyte cultures via CMA activation. Panels (A,B): Pioglitazone and metformin at pharmacological concentrations significantly increased AMPK Thr172 phosphorylation, (pioglitazone in human primary hepatocytes: from 0.43 ± 0.04 to 1.18 ± 0.26, P = 0.04; metformin in human primary hepatocytes: from 0.43 ± 0.04 to 0.94 ± 0.12, P = 0.005; pioglitazone in rat primary hepatocytes: from 0.81 ± 0.11 to 1.87 ± 0.31, P = 0.03; metformin in rat primary hepatocytes: from 0.81 ± 0.11 to 1.50 ± 0.14, P = 0.01). Panels (C,D): Pioglitazone and metformin at pharmacological concentrations significantly increased LAMP2A protein expression (pioglitazone in human primary hepatocytes: from 2.16 ± 0.21 to 3.40 ± 0.37, P = 0.02; metformin in human primary hepatocytes: from 2.16 ± 0.21 to 3.30 ± 0.36, P = 0.04; pioglitazone in rat primary hepatocytes: from 2.91 ± 0.70 to 4.92 ± 0.90, P = 0.02; metformin in rat primary hepatocytes: from 2.91 ± 0.70 to 5.62 ± 1.11, P = 0.04). Panels (E,F): Pioglitazone and metformin at pharmacological concentrations significantly decreased Plin2 protein expression (pioglitazone in human primary hepatocytes: from 0.67 ± 0.13 to 0.38 ± 0.08, P = 0.02; metformin in human primary hepatocytes: from 0.67 ± 0.13 to 0.30 ± 0.06, P = 0.02; pioglitazone in rat primary hepatocytes: from 0.65 ± 0.11 to 0.41 ± 0.11, P = 0.03; metformin in rat primary hepatocytes: from 0.65 ± 0.11 to 0.39 ± 0.06, P = 0.02). Data are expressed as mean ± SEM (n = 5 controls).

Human and rat primary hepatocytes were cultured for 24 h in the presence of high levels of oleic acid, glucose and insulin to induce insulin resistance. Figure 6 shows that both metformin and pioglitazone at pharmacological concentrations decreased Plin2 expression by promoting AMPK phosphorylation and consequent activation of CMA. Indeed, AMPK phosphorylation was increased after pioglitazone or metformin exposure (pioglitazone in human primary hepatocytes: from 0.43 ± 0.04 to 1.18 ± 0.26, P = 0.04; metformin in human primary hepatocytes: from 0.43 ± 0.04 to 0.94 ± 0.12, P = 0.005; pioglitazone in rat primary hepatocytes: from 0.81 ± 0.11 to 1.87 ± 0.31, P = 0.03; metformin in rat primary hepatocytes: from 0.81 ± 0.11 to 1.50 ± 0.14, P = 0.01) (Fig. 6, Panels A,B).

Pioglitazone or metformin increased LAMP2A expression (pioglitazone in human primary hepatocytes: from 2.16 ± 0.21 to 3.40 ± 0.37, P = 0.02; metformin in human primary hepatocytes: from 2.16 ± 0.21 to 3.30 ± 0.36, P = 0.04; pioglitazone in rat primary hepatocytes: from 2.91 ± 0.70 to 4.92 ± 0.90, P = 0.02; metformin in rat primary hepatocytes: from 2.91 ± 0.70 to 5.62 ± 1.11, P = 0.04) (Fig. 6, Panels C,D).

In contrast, pioglitazone or metformin significantly decreased Plin2 expression (pioglitazone in human primary hepatocytes: from 0.67 ± 0.13 to 0.38 ± 0.08, P = 0.02; metformin in human primary hepatocytes: from 0.67 ± 0.13 to 0.30 ± 0.06, P = 0.02; pioglitazone in rat primary hepatocytes: from 0.65 ± 0.11 to 0.41 ± 0.11, P = 0.03; metformin in rat primary hepatocytes: from 0.65 ± 0.11 to 0.39 ± 0.06, P = 0.02) (Fig. 6, Panels E,F).

**Figure 7:**
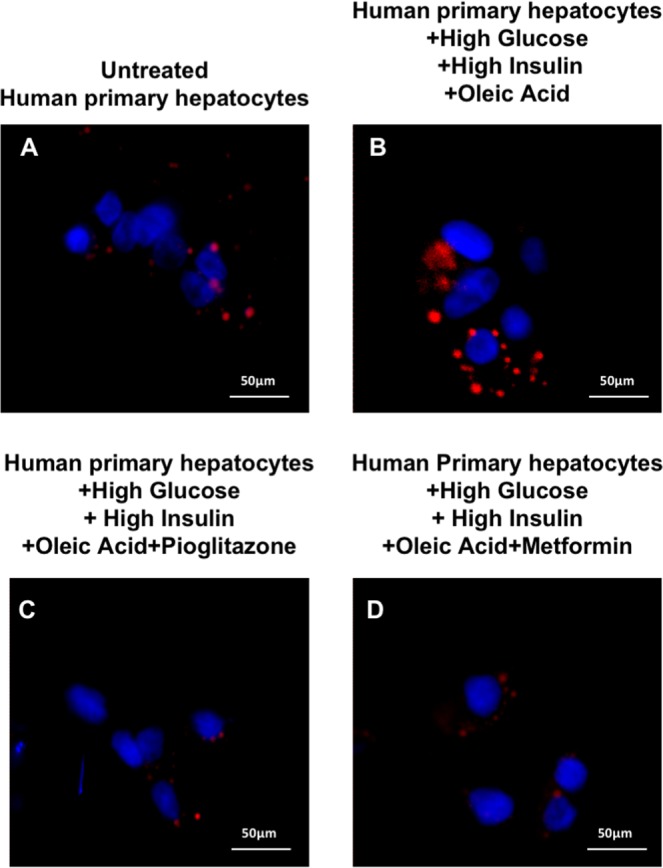
Pioglitazone or metformin decreased lipid droplet accumulation in human primary hepatocyte cultures. Panel (A): Nile Red staining of untreated hepatocyte primary culture. Panel (B): Nile Red staining of primary hepatocytes cultured for 24 h in the presence of high levels of oleic acid, glucose and insulin. Panel (C): Nile Red staining of primary hepatocytes cultured for 24 h in the presence of high levels of oleic acid, glucose, insulin and pioglitazone. Panel (D): Nile Red staining of primary hepatocytes cultured for 24 h in the presence of high levels of oleic acid, glucose, insulin and metformin.

Moreover, Nile Red staining shows a reduction in lipid droplet accumulation after both pioglitazone (Fig. 7, Panel C) and metformin (Fig. 7, Panel D) exposure in human hepatocytes primary cultures.

## Discussion

Non-alcoholic fatty-liver disease (NAFLD) is currently the most frequent liver disorder in the Western world^1^. Diabetes is a major risk factor for the development of NAFLD. Currently, there are no FDA-approved drugs specifically tailored for NAFLD or NASH. Bariatric/metabolic surgery is effective in reversing the hepatic features of NAFLD and NASH, as shown by retrospective or prospective but non randomized studies^11^. However, the mechanism of action of bariatric/metabolic surgery remains elusive.

In the present study, we provide evidences both in rodents and in humans that after SG there is a net increase of phosphorylated AMPK and LAMP2A expression, causing a decrease in Plin2 expression and a reduction in LDs accumulation in both liver and circulating monocytes. In addition, we observed an increased phosphorylation of Akt Ser473, GSK3αβ Ser21/9 and FoxO1 Thr24 and an increased glycogen deposition in liver and monocytes of both rodents and humans after SG.

We also report that fat accumulation in monocytes was well comparable to that in hepatocytes, suggesting that Plin2 levels in monocytes might be a non-invasive marker for the diagnosis of NAFLD.

The changes in phosphorylation of proteins of interest were independent of changes in body weight both in humans and in rats suggesting a weight-independent mechanism of action of SG. Most beneficial effects of sleeve gastrectomy are, in fact, achieved in a weight-independent manner as shown by Frühbeck^21^, although the mechanism of action is not completely elucidated. One of the pathways affected by caloric restriction is the AMPK one. AMPK controls glucose disposal by inhibiting liver gluconeogenesis and increasing muscle glucose uptake^22–25^. Targeting hepatic AMPK with an AMPK-specific small-molecule activator reduces liver steatosis and improves glucose disposal in obese mice^26,27^. Recently, Garcia *et al*.^28^ showed that liver-specific AMPK activation in DIO mice protects against liver steatosis.

Our data show a net increase of AMPK phosphorylation after SG in both hepatocytes and monocytes either in rodents or in humans. AMPK phosphorylation is a crucial step for the binding of Plin2 to LAMP2A that leads to an increased Plin2 catabolism and lipid droplets exposure to lypolysis^19^. Indeed, after SG we found a significant reduction of Plin2 expression and lipid droplets accumulation.

Bariatric/metabolic surgery procedures that reduce caloric intake, including SG, have shown high rates of remission of type 2 diabetes mostly due to the improvement of insulin sensitivity^7^. In fact, here we report a significant increase of Akt Ser473, GSK3αβ Ser21/9 and FoxO1 Thr24 phosphorylation in both liver and monocytes, which points towards an improved hepatic insulin sensitivity with reduced gluconeogenesis and increased glycogen synthesis. Indeed, the inhibition of GSKαβ activity through its phosphorylation leads to increased glycogen synthesis, while the inhibition of FoxO1 activity, again through its phosphorylation, reduces gluconeogenesis^29^.

These results are supported by *in vitro* data showing that overexpression of Plin2, using a human-Plin2 plasmid in primary hepatocyte and monocyte cultures, significantly affects insulin signaling decreasing Akt Ser473, GSK3αβ Ser21/9 and FoxO1 Thr24 phosphorylation.

Several studies have shown that AMPK is activated by two major antidiabetic drugs, metformin and pioglitazone^19,30^. We report that treatment of primary hepatocytes with pioglitazone or metformin reduces Plin2 expression, LDs accumulation and improves insulin signaling.

When insulin binds rodent or human hepatocytes it is internalized and preferentially associated with lysosomal structures^20,31^. The upregulation of LAMP2A we found might, thus, reflect the improvement of hepatic insulin resistance after sleeve gastrectomy that was shown by the significant reduction of HOMA-IR values.

The lack of post-surgery liver biopsies and the lack of a pair-fed group of rats represent two limitations of our study, although our results in humans are supported by the similar findings associated with SG in rodents. We showed, in fact, a high correlation between LDs and Plin2 content in the liver and in monocytes that were available also after SG. Furthermore, the surrogate marker HOMA-IR, that has been shown to be highly correlated with hepatic insulin resistance as assessed by the gold standard euglycemic hyperinsulinemic clamp^32^, was used to assess hepatic insulin resistance.

**Figure 8:**
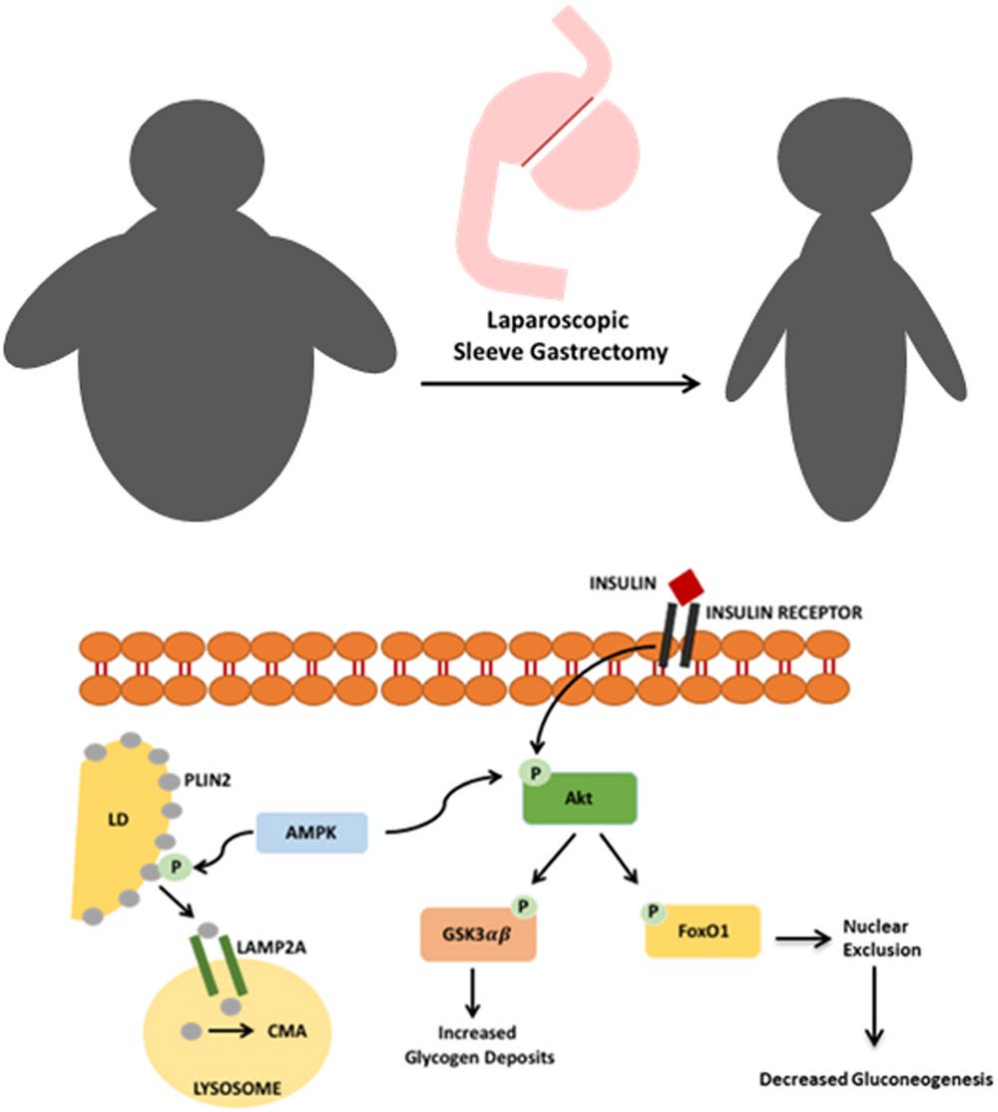
Proposed mechanism of NAFLD and insulin resistance reversal after Sleeve Gastrectomy. Excessive caloric intake, associated with NAFLD and obesity, reduces AMPK activation, causing a decrease of Plin2 catabolism mediated by chaperone-mediated autophagy leading to ectopic fat deposition increase and simultaneously to hepatic insulin resistance. After SG, the energy imbalance due to reduced caloric intake causes an increase in AMPK phosphorylation, a crucial step in Plin2-LAMP2A binding, leading to an enhanced Plin2 autophagy that exposes LD triglycerides to intracellular lipases. In addition, AMPK increased phosphorylation improves insulin signaling and promotes glycogen synthesis.

In conclusion, we propose that excessive caloric intake, which is associated with NAFLD and obesity, reduces AMPK activation, causing a decrease of Plin2 catabolism mediated by chaperone-mediated autophagy that leads to an increase of ectopic fat deposition and simultaneously to hepatic insulin resistance. After SG, the energy imbalance due to reduced caloric intake causes an increase in AMPK phosphorylation, a crucial step in Plin2-LAMP2A binding, leading to an enhanced Plin2 autophagy that exposes LD triglycerides to intracellular lipases. In addition, AMPK increased phosphorylation improves insulin signaling and promotes glycogen synthesis (Fig. 8).

## Methods

### Animal model

Thirty Wistar rats of both sexes aged 10 weeks were housed individually in a controlled room at 22 °C with a 12-h day/night cycle (lights on 0700–1900 h). The animals received a purified tripalmitin-based High fat liquid diet (HFD) ad libitum (Rieper AG, Bolzano, Italy) supplying 71% of energy from saturated fat, although corn oil (1.9/100 g diet) was present in order to prevent essential fatty acid deficiency, 20% from carbohydrate comprising cornstarch and sucrose (2:1 weight for weight) and 10% from protein. The HFD was continued for 10 weeks before and 15 weeks after the operation. The animals randomly underwent SG or sham operation. Survival rates were 90% after sham operation, and 75% after SG. All experimental procedures were approved by the Catholic University of Rome Institutional Animal Care Committee and all methods were performed in accordance with the relevant guidelines and regulations^33^.

### Interventions

The rats were anesthetized using ketamine (75 mg/kg intramuscularly) and xylazine (10 mg/kg intramuscularly). Ten milliliters of sterile 0.9% NaCl were administered subcutaneously before surgery. Access to the peritoneal cavity was obtained by 3-cm laparotomy^33^.

### Vertical sleeve gastrectomy

A laparotomy incision was made in the abdominal wall and the stomach was isolated outside the abdominal cavity and placed on saline-moistened gauze pads. The gastric to spleen connections were released along the greater curvature. The lateral 80% of the stomach was excised leaving a tubular gastric remnant in continuity with the esophagus and with the pylorus and duodenum. After the operation, the abdominal wall was closed in layers^33^.

In sham-operated rats, a midline laparotomy was performed, and the stomach was exposed and gently manipulated. The abdominal cavity was kept open for the same amount of time required to perform the other operations. A 1-cm gastrotomy was performed and then closed as in the SG group.

### Postoperative care

At the end of the surgical procedures, all rats received sterile 0.9% NaCl 10 mL i.p. and 10 mL s.c. to maintain hydration during healing. The animals received ketoprofen 5 mg/kg as an analgesic. They were placed on a heated mat until they recovered and then were returned to their home cages. The rats were allowed to drink purified water for 12 h after surgery, and a liquid diet containing 5% glucose and 0.2% KCl was provided for the next 48 h. Thereafter, they received the HFD until 15 weeks after surgery^34^.

### Oral glucose tolerance test

The oral glucose tolerance test (OGTT) was performed at kill. Animals were fasted overnight and then received a 50% D-glucose solution (1 g/kg body weight) by oral gavage. Blood was collected from the tail vein for measurement of glucose and insulin concentrations at 0, 15, 30, 60, 90,120 and 180 min at the end of the study. After centrifugation, plasma was divided into appropriate subsamples and stored at −20 °C until analyses.

### Analytical methods

Blood glucose levels were analyzed by glucometer (Accu-Chek, Roche Diagnostics Division, Grenzacherstrasse, Switzerland). Serum insulin was measured by a rat insulin ultrasensitive ELISA (Biovendor GmbH, Kassel, Germany), with a sensitivity of 0.025 ng/ml and an intra- and inter-assay precision of 10%.

### Hepatocytes isolation

Hepatocytes were isolated by collagenase perfusion^35^ of the liver. Cells were cultured for 24 hours in DMEM with 10% FBS.

## Human Studies

### Study population

We enrolled 15 obese subjects with NAFLD of both sexes (age 39.7 ± 2.1) before and one year after laparoscopic SG. Eight subjects who underwent laparoscopic elective cholecystectomy, but otherwise in healthy conditions, served as controls. Venous blood samples were collected, after 12 hours fasting, at the time of the patients’ enrolment and one year after SG.

Comprehensive history was obtained from all subjects. Physical exam, including height, weight and anthropometric measurements were conducted. Body mass index (BMI) was calculated by dividing the weight (kg) by height (cm). A detailed medication list was obtained. All participants underwent biochemical tests: ALT, AST, fasting glucose, insulin, triglycerides, HDL and LDL-cholesterol.

Exclusion criteria were^1^: regular and/or excessive alcohol use (>20 g of alcohol per day for women and >30 g of alcohol per day for men)^2^; clinically evidence of secondary NALFD due to iatrogenic gastrointestinal disorder or immune deficiency (HIV) infection^3^; clinically evidence of non-NAFLD liver disease including hepatitis B or C, hemochromatosis^4^, Wilson disease^5^, glycogen storage disease^6^, alpha-1antitrypsin deficiency^7^, autoimmune hepatitis^8^, cholestatic liver disease^9^, presence of major cardiovascular, gastrointestinal, or respiratory disease, or any hormonal disorders^10^, clinically evidence of decompensated liver disease (Child-Pugh score >7 points)^11^, active substance abuse^12^, significant systemic illnesses^13^, pregnant status^14^, type 2 diabetes.

The study was approved by the Ethical Committee of the University of Rome “La Sapienza” and the patients signed an informed consent prior the study enrolment; in addition, they signed a specific informed consent for the surgical operations and all methods were performed in accordance with the relevant guidelines and regulations

### Human sleeve gastrectomy

Sleeve Gastrectomy (SG) consists in a vertical resection of the stomach in order to obtain a residual gastric capacity ranged from 60 to 80 ml. The procedures were carried out by laparoscopy using 5 trocars. The resection was performed using linear stapler with sequential cartridges alongside an oro-gastric calibration tube placed against the lesser curvature. Resection was begun 4 to 6 cm proximal to the pylorus and was continued up-ward to the angle of His with complete excision of the fundus, part of the body and antrum.

During the first month after surgery patients had a semiliquid diet with about 850 kcal per day for the first month and thereafter they gradually introduced solid foodstuffs having a free diet.

### Liver histology

Human liver biopsy material, obtained during the operation, was mounted on archival slides originally prepared from 4% formaldehyde-fixed paraffin-embedded tissue and stained with hematoxylin-eosin to evaluate the percentage of steatosis. Blind analysis of biopsy specimens was performed by a liver pathologist (CG).

We used the Brunt classification^36^ for the histological grading and staging of NAFLD/NASH. In particular, steatosis was graded as 0, none (<1%); 1, 1–25%; 2, 26–50%; 3, 51–75%; and 4, >75% amount of fat in the lobules. Inflammation was graded as 1, mild (scattered lymphocytes or small clusters within portal tracts and lobules); 2, moderate (same as grade 1 but with a larger portal and lobular infiltration); and 3, severe (the same as grade 2 but with more intense inflammation). Fibrosis was staged as 0 if absent, 1 if centrilobular pericellular; 2, if periportal and pericellular; 3, bridging fibrosis; and 4, cirrhosis.

### Insulin sensitivity

Hepatic insulin sensitivity was measured by the homeostasis model assessment (HOMA-IR) as fasting insulin (μU/ml) x fasting glucose (mmol/ml)/22.5^37^.

### Lipid staining and glycogen storage

Periodic acid Schiff staining was used to evaluate glycogen storage. Slides were fixed 20 minutes with 4% formaldehyde, stained in Periodic Acid Solution for 5 minutes and in Schiff’s Reagent for 15 minutes. Counterstain was performed with Hematoxylin solution.

Oil Red O was performed to assess intracellular lipid accumulation. Slides were fixed overnight with 4% formaldehyde, stained with Oil Red O solution for 1 hour. Counterstain was performed with Hematoxylin solution. Photographs of stained sections were taken with an optical microscope (ZEISS Primo Star HAL/LED).

### Monocytes isolation

PBMCs were obtained from whole blood samples by standard gradient centrifugation over Ficoll-Hypaque. PBMCs were washed and monocytes were isolated following Pan Monocyte Isolation Kit instruction.

### Human primary hepatocytes isolation

Hepatocytes from obese, NAFLD subjects and healthy subjects were isolated as described elsewhere^38^. Briefly, tissue was diced and washed in HBSS to remove excess blood. Tissue was transferred to a 50 ml tube containing EGTA buffer (HBSS, 0.5 micromol/L EGTA, 0.5% BSA) and agitated at 100 rpm in a water bath with shaking bed for 10 min at 37 °C. Tissue was then placed in digestion buffer (HBSS, 0.05% collagenase IV, 0.5% fatty acid free BSA, 10 micromol/L CaCl2) and agitated in a water bath with shaking bed for 30 min at 37 °C. Supernatant was collected and filtered through 100 μm cell strainer and the cell suspension centrifuged at 80 g for 5 min at 4 °C and the supernatant discarded. Cells were cultured in DMEM in collagen coated dish at 37 °C and 5%CO_2_.

### Antibodies and reagents

Antibodies against phospho-Akt (Ser473), phospho-GSK3αβ (Ser21/9), pospho-AMPK(Thr172) and phospho-FoxO1 (Thr24) were obtained from Cell Signaling Technology (Danvers, MA). Plin2 antibody was obtained from LS-BIO (Seattle, WA). LAMP2A was obtained from Thermo Fisher scientific (Waltham, MA). β-actin antibody was obtained from Santa Cruz Technology (Santa Cruz, CA). Period Acid, Schiff’s reagent, Oil red O, Nile red, DAPI and Hematoxylin were obtained from Merck (Darmstadt, DE).

### Western blot analysis

Monocytes and liver biopsies were homogenized in RIPA buffer containing a cocktail of protease inhibitors. Homogenates were cleared by centrifugation (19.000 g; 30 min, 4 °C). Protein content was determined using Bradford Protein Assay. Protein lysates (30 μg) were separated on 10% SDS-PAGE and transferred on PVDF membrane^33,39^. Membranes were probed overnight with Plin2, phospho-AktSer473, GSK3αβ Ser21/9, FoxO1 Thr24, AMPK Thr172, LAMP2A, and βActin. Membranes with phospho-antibodies were stripped for 30 min at 56 °C and reprobed overnight with total Akt, GSK3α-β, AMPK and FoxO1respectively. Detection and analysis were performed respectively with Chemidoc XRS Image system and Image Lab 5.0 software (Bio-Rad Laboratories, Hercules, CA)^33,39^. Plin2 and LAMP2A were normalized with βActin while phospho-AktSer473, GSK3αβ Ser21/9, FoxO1 Thr24 and AMPK Thr172 were normalized with total Akt, GSK3α-β, FoxO1, AMPK respectively and expressed as a phospho-protein/total protein ratio.

## *In vitro* Study

### Lipid droplet accumulation

Primary hepatocytes and monocytes were stimulated with Oleic Acid (400 µmol/L) for 24 hours and stained with Nile Red (100 ng/mL). Flow cytometric analysis and confocal imaging were conducted to quantify lipid droplets. Flow cytometric analysis was conducted with FC 500 (Beckman Coulter, Brea, CA) and data were analyzed with Kaluza software (Beckman Coulter, Brea, CA). Photographs were taken using confocal microscope Spinning Disk; Crest X-Light Confocal Imager (Germany) and MetaMorph Microscopy Automation & Image Analysis Software (Molecular Devices) was used to analyze images.

Quantitative data from flow cytometry analysis were used to assess the cellular content of LDs stained with Nile Red and used for the Bland Altman plot.

### Flow cytometry

Monocytes and primary hepatocytes were washed and resuspended in PBS containing 5% FBS. Cells were fixed, permeabilized and stained for Plin2 using AlexaFluor 488 as secondary antibody, with CD14 to identify monocytes population and GLUT2 to identify hepatocytes. Flow cytometric analysis was conducted with FC 500 (Beckman Coulter, Brea, CA) and data were analyzed with Kaluza software (Beckman Coulter, Brea, CA).

### Plin2 transfection

Primary hepatocytes and monocytes were transfected with human PLIN2 plasmid using Nucleofector™ Kits and following manufactures instruction. As a positive control for transfection we used 2 μg pmaxGFP® Vector. Untrasfected cells and cells transfected with human Plin2 plasmid were culture for 6 hours. To evaluate the efficiency of transfection and Plin2 overexpression cells were analyzed with flow cytometry. To asses Plin2 overexpression effect on AktSer473, GSK3αβ Ser21/9 and FoxO1 Thr24 cells were analyzed by western blot.

### Pioglitazone and metformin exposure

Primary hepatocytes were incubated for 24 hrs with insulin (100 nmol/L), Glucose (25 mmol/L), Oleic Acid (400 µmol/L) and with or without pioglitazione (25 µmol/L) or metformin (2 mmol/L). Western blot analysis were performed to assess AMPK Thr172, LAMP2A and Plin2 expression.

### Lipid staining

Primary hepatocytes were incubated for 45 minutes at 37 °C and 5%CO_2_with Nile Red (100 ng/mL). Nuclear staining was performed with DAPI.

Photographs were taken using confocal microscope Spinning Disk; Crest X-Light Confocal Imager (Germany) and MetaMorph Microscopy Automation & Image Analysis Software (Molecular Devices) was used to analyze images.

### Statistical analysis

Data were expressed as means ± s.e.m. unless specified otherwise. Statistical analyses (SPSS version 13) were performed using Mann-Whitney or Wilcoxon tests and repeated measures test with Bonferroni’s correction or Dunn’s Multiple Comparison test where appropriate. Differences were considered statistically significant at P ≤ 0.05.

## Supplementary information

Supplementary Information

## Data Availability

The authors declare that all the data supporting the findings of this study are available in the manuscript, figures and supplementary information files.
