# Publisher Correction: New insight into the mechanisms of ectopic fat deposition improvement after bariatric surgery

**DOI:** 10.1038/s41598-020-58961-0

**Published:** 2020-02-12

**Authors:** Giulia Angelini, Lidia Castagneto Gissey, Giulia Del Corpo, Carla Giordano, Bruna Cerbelli, Anna Severino, Melania Manco, Nicola Basso, Andreas L. Birkenfeld, Stefan R. Bornstein, Alfredo Genco, Geltrude Mingrone, Giovanni Casella

**Affiliations:** 1 Fondazione Policlinico Universitario A. Gemelli IRCCS, Rome, Italy and Università Cattolica del S. Cuore, Rome, Italy; 2 Department of Surgical Sciences, Sapienza University of Rome, Rome, Italy; 3 Department of Radiological, Oncological and Pathological Sciences, Sapienza University of Rome, Rome, Italy; 4 Research Unit for Multi-factorial Diseases, Obesity and Diabetes, Istituti di Ricovero e Cura a Carattere Scientifico, Bambino Gesù Children’s Hospital, Rome, Italy; 5 Department of Medicine III, Universitätsklinikum Carl Gustav Carus an der Technischen Universität Dresden, Dresden, Germany; 6 Paul Langerhans Institute Dresden of the Helmholtz Center Munich at University Hospital and Faculty of Medicine, TU Dresden, Dresden, Germany; 7 Diabetes and Nutritional Sciences, King’s College London, London, United Kingdom; 8 Deutsches Zentrum für Diabetesforschung, DZD e.V., Neuherberg, Germany

Correction to: *Scientific Reports* 10.1038/s41598-019-53702-4, published online 21 November 2019

In the original version of this Article, the author Lidia Castagneto Gissey was incorrectly indexed. This error has now been corrected.

