# Supplementary material for: New insight into the mechanisms of ectopic fat deposition improvement after bariatric surgery
Source: Sci Rep. 2019 Nov 21;9:17315. doi: 10.1038/s41598-019-53702-4 (PMC6872729; doi:10.1038/s41598-019-53702-4)

**New insight into the mechanisms of ectopic fat deposition improvement after bariatric surgery**

^1^Giulia Angelini, ^2^Lidia Castagneto Gissey, ^2^Giulia Del Corpo, ^3^Carla Giordano, ^3^Bruna Cerbelli, ^1^Anna Severino, ^4^Melania Manco, ^2^Nicola Basso, ^5,6,7,8^Andreas L. Birkenfeld ^5,6,7,8^Stefan R. Bornstein, ^2^Alfredo Genco, ^1,7^Geltrude Mingrone, ^2^Giovanni Casella

^1^Fondazione Policlinico Universitario A. Gemelli IRCCS, Rome, Italy and Università Cattolica del S. Cuore, Rome, Italy.

^2^Department of Surgical Sciences, Sapienza University of Rome, Rome, Italy

^3^Department of Radiological, Oncological and Pathological Sciences, Sapienza University of Rome, Italy

^4^ Research Unit for Multi-factorial Diseases, Obesity and Diabetes, Istituti di Ricovero e Cura a Carattere Scientifico, Bambino Gesù Children's Hospital, Rome, Italy.

^5^Department of Medicine III, Universitätsklinikum Carl Gustav Carus an der Technischen Universität Dresden, Dresden, Germany.

^6^ Paul Langerhans Institute Dresden of the Helmholtz Center Munich at University Hospital and Faculty of Medicine, TU Dresden, Dresden, Germany

^7^Diabetes and Nutritional Sciences, King's College London, London, United Kingdom.

^8^ Deutsches Zentrum für Diabetesforschung, DZD e.V., Neuherberg, Germany

Corresponding author:

Professor Giovanni Casella

Department of Surgery

University of Rome “La Sapienza”

Email: giovanni.casella@uniroma1.it

**Table 1.** Statistical data of Plin2-clinical parameters linear regression in monocytes

| Subjects | R^2^ | P value | |
| --- | --- | --- | --- |
| HOMA-IR |  | |  |
| Before Sleeve Gastrectomy | 0.55 | | 0.002 |
| After Sleeve Gastrectomy | 0.71 | | <0.0001 |
| Controls | 0.79 | | 0.003 |
|  |  | |  |
| LDL-Cholesterol |  | |  |
| Before Sleeve Gastrectomy | 0.42 | | 0.008 |
| After Sleeve Gastrectomy | 0.38 | | 0.01 |
| Controls | 0.69 | | 0.01 |
|  |  | |  |
| AST |  | |  |
| Before Sleeve Gastrectomy | 0.40 | | 0.02 |
| After Sleeve Gastrectomy | 0.45 | | 0.005 |
| Controls | 0.87 | | 0.0002 |
|  |  | |  |
| ALT |  | |  |
| Before Sleeve Gastrectomy | 0.47 | | 0.004 |
| After Sleeve Gastrectomy | 0.33 | | 0.02 |
| Controls | 0.59 | | 0.01 |
|  |  | |  |


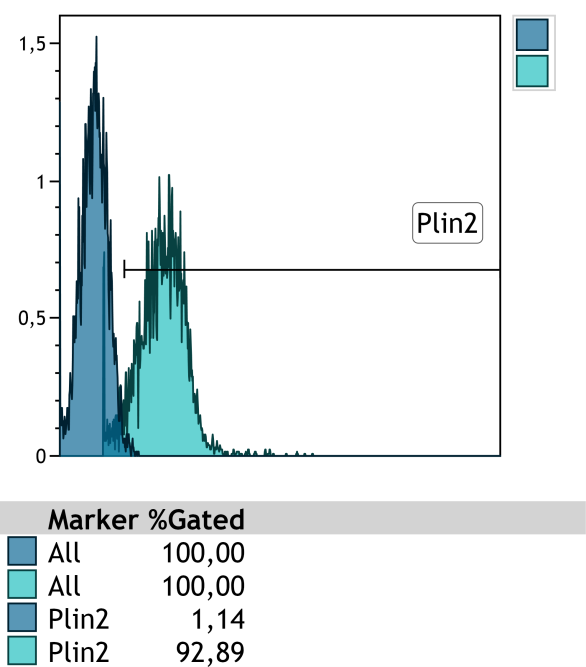

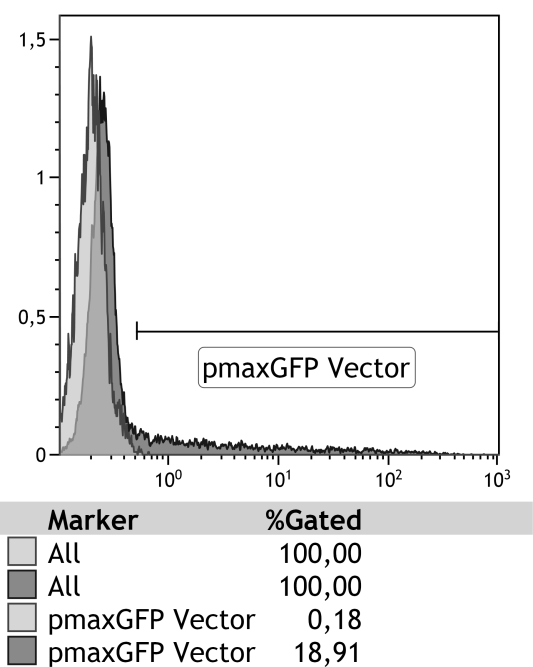

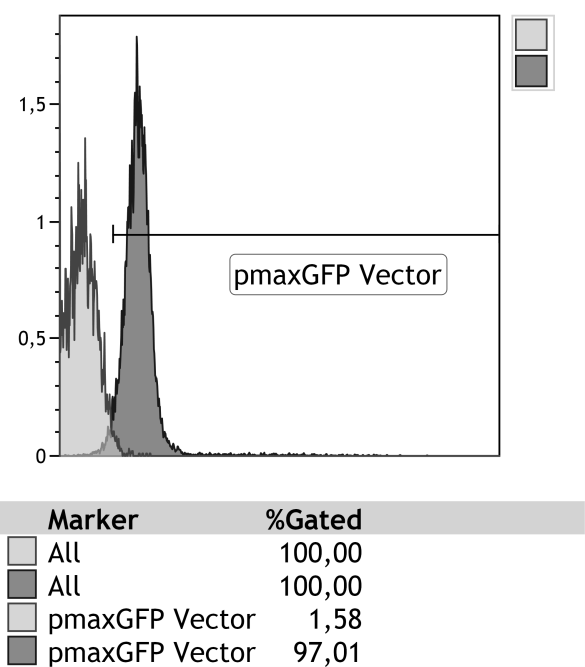

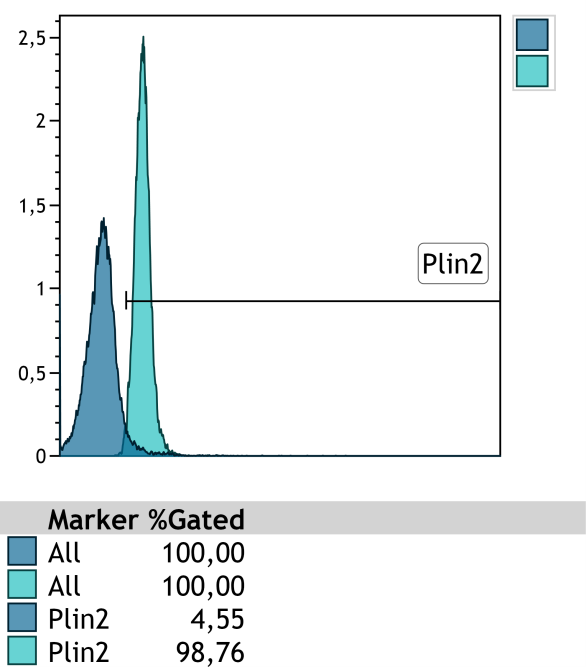

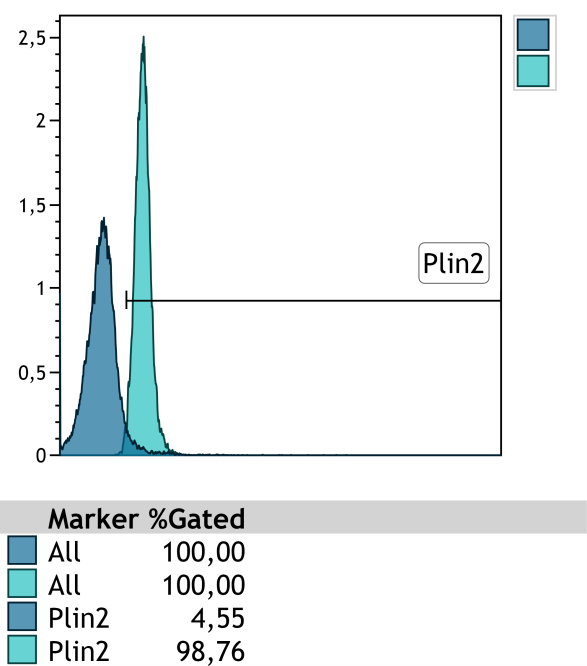

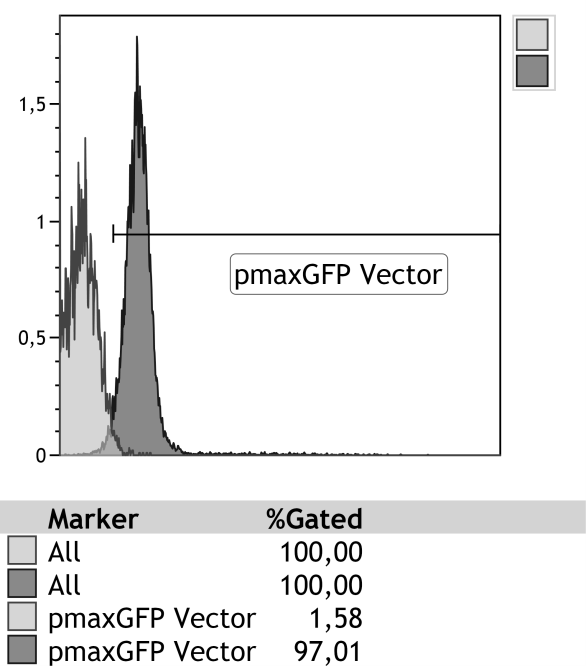


NT

NT

+ pmaxGFP Vector

+ Plin2-GFP Vector

A

B

C

D

**Primary Cultures**

**of monocytes**

**Primary cultures**

**of hepatocytes**


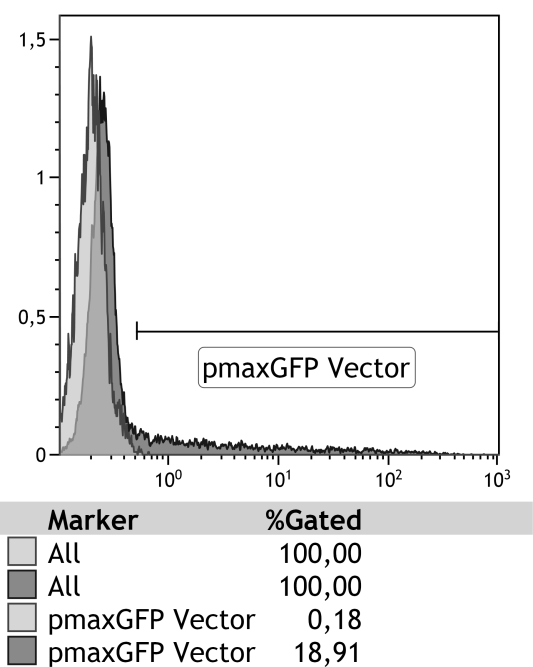

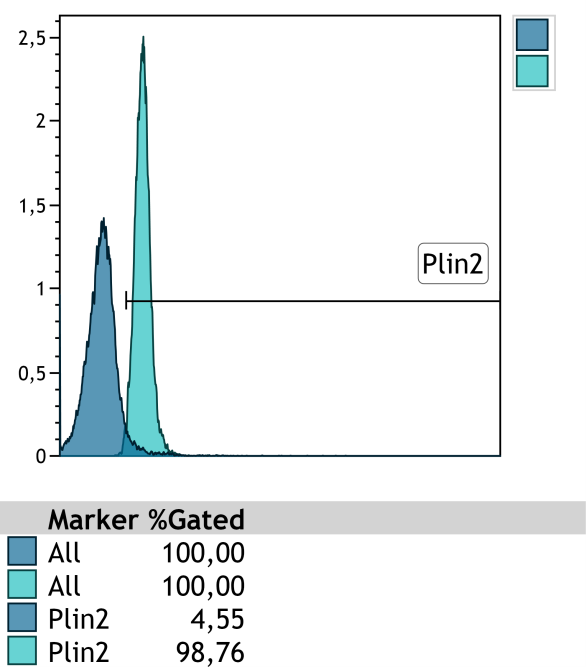

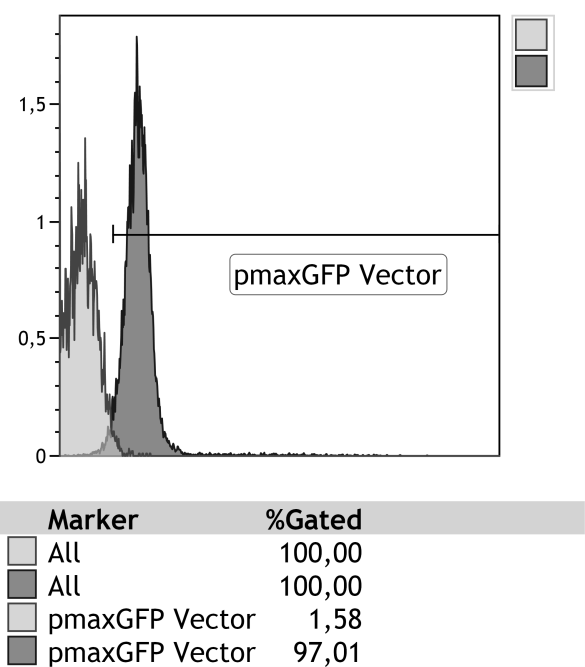

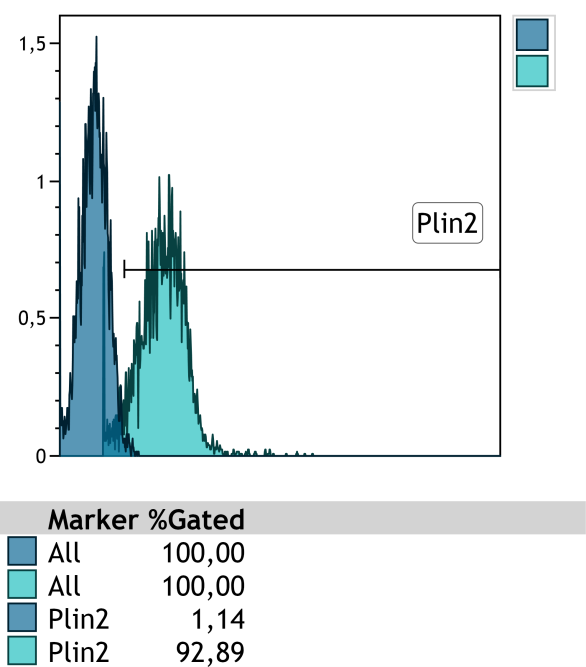


**Supplementary Figure 1. Cell transfection**

**Panels A and C:** Cell transfection efficiency quantified with pmaxGFP vector.

**Panel B and D:** Plin2 quantification by flow cytometry after transfection.

**Uncropped Gels**


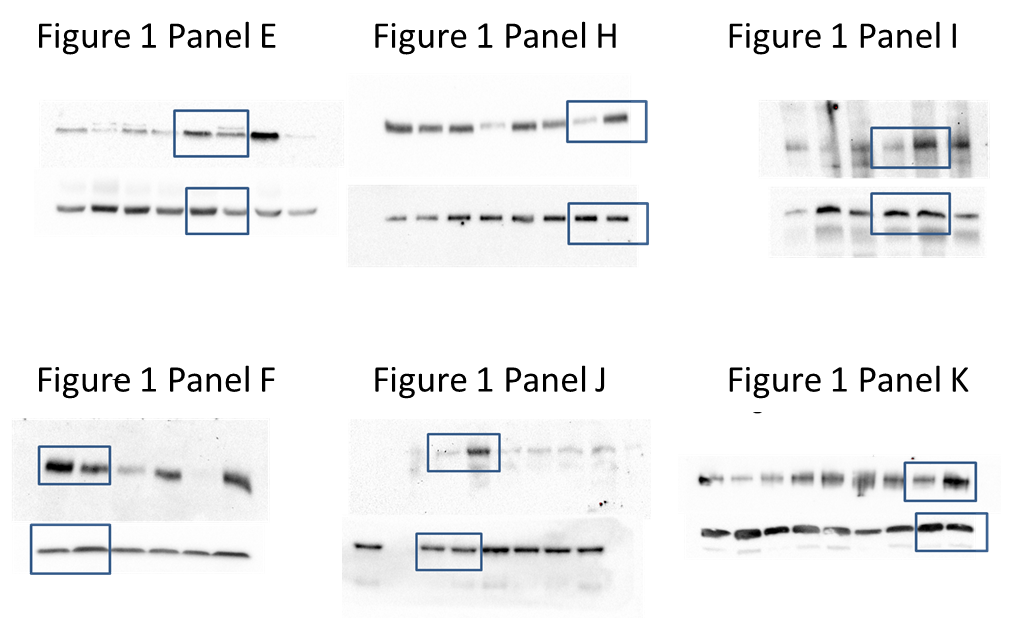


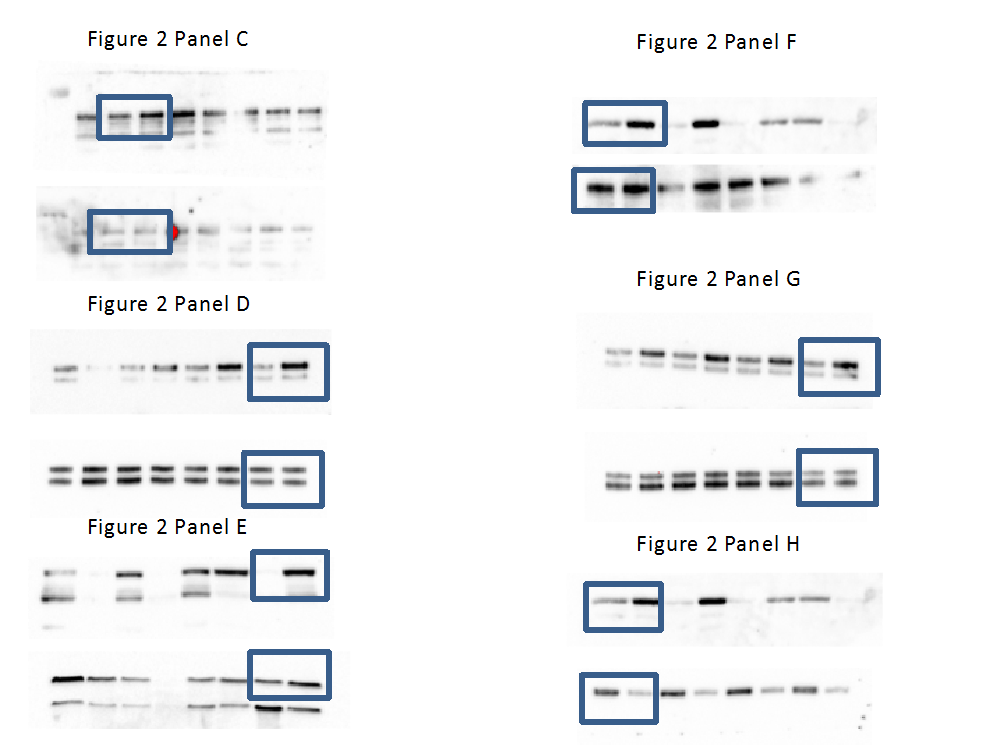


Figure 2 Panel C

Figure 2 Panel F

Figure 2 Panel D

Figure 2 Panel G

Figure 2 Panel E

Figure 2 Panel H


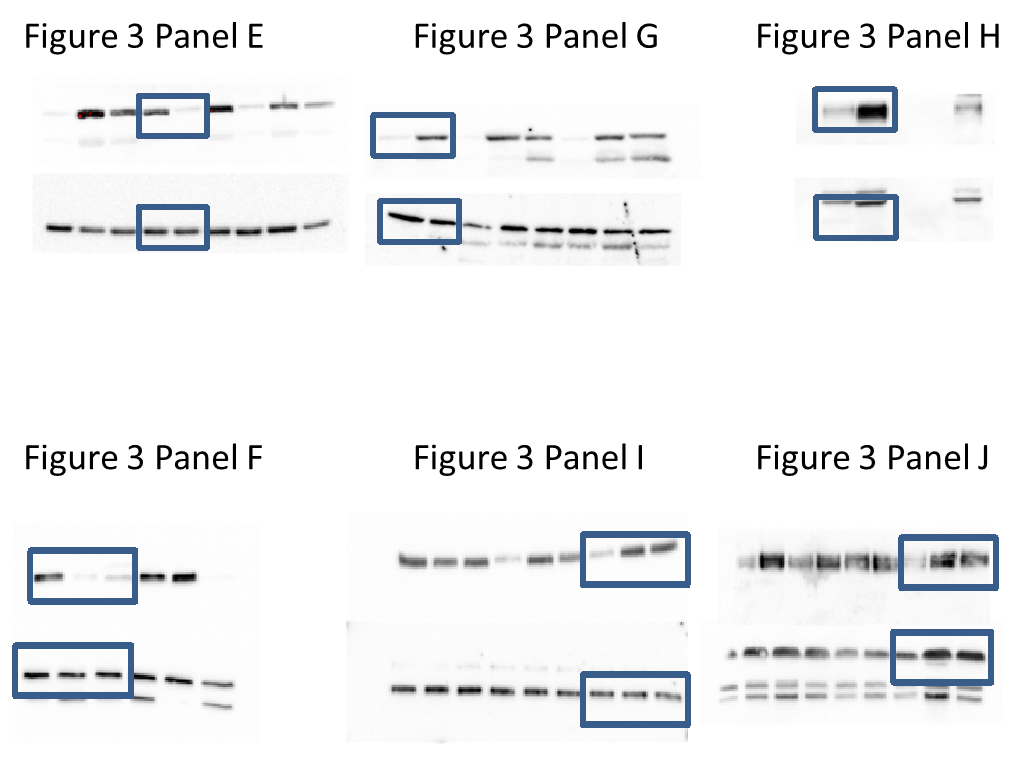


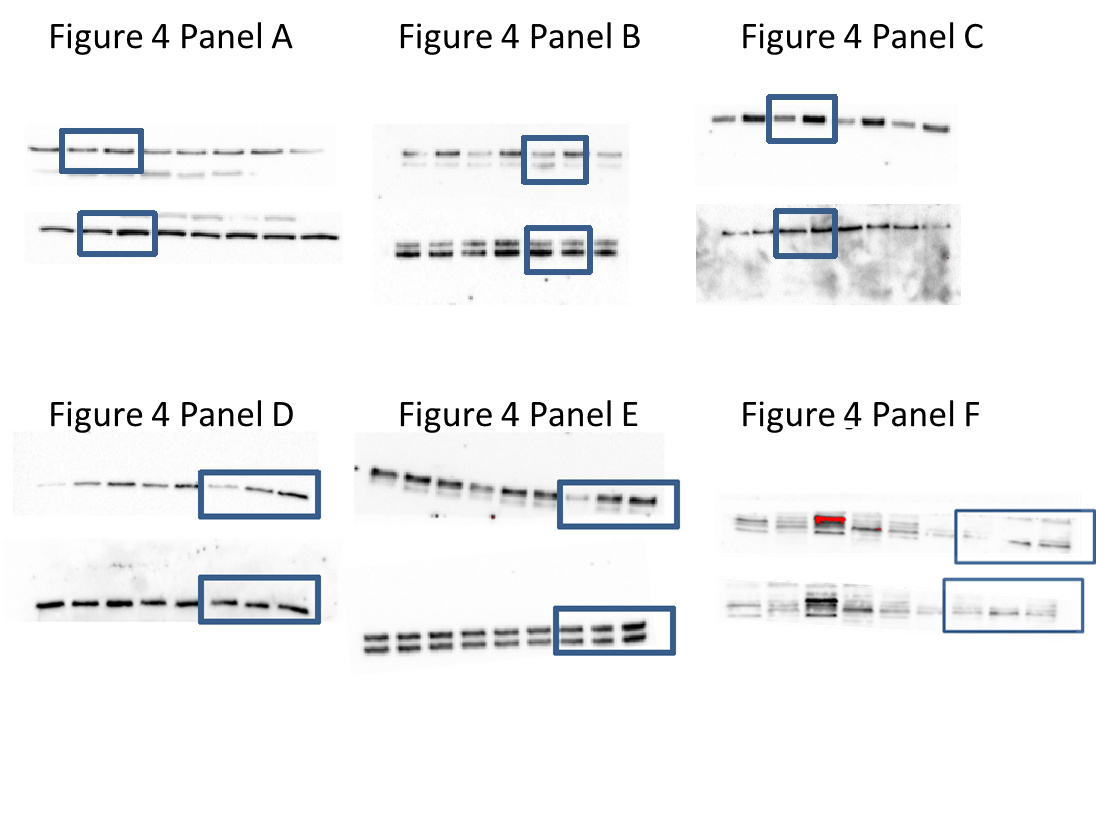


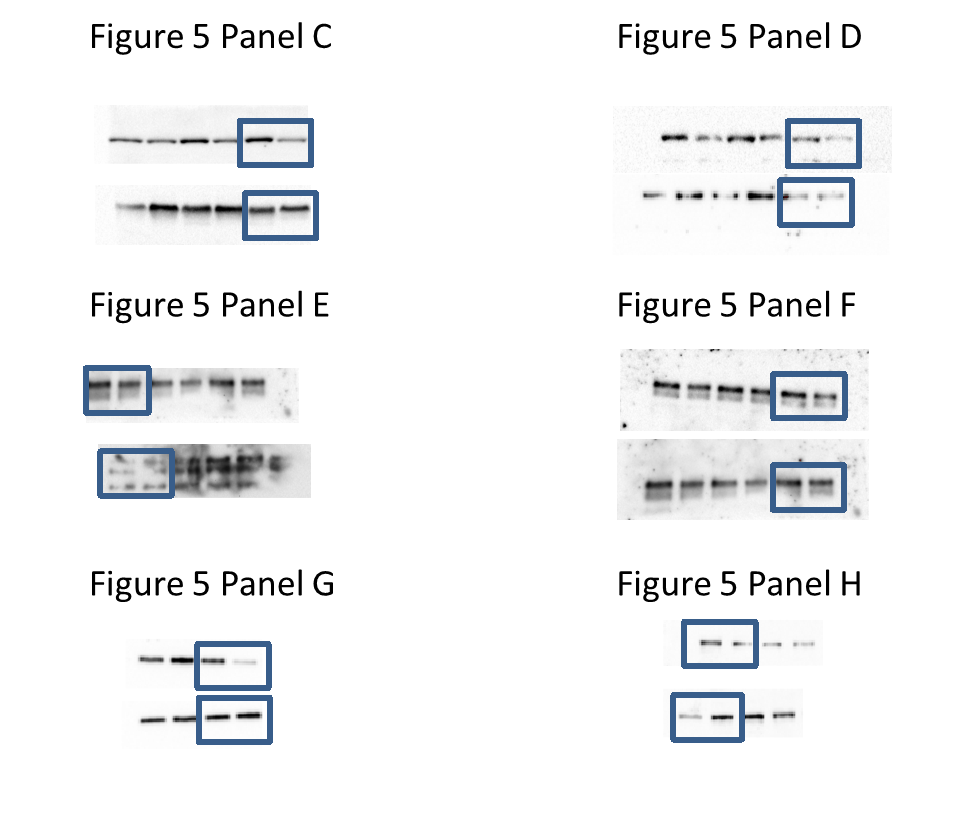


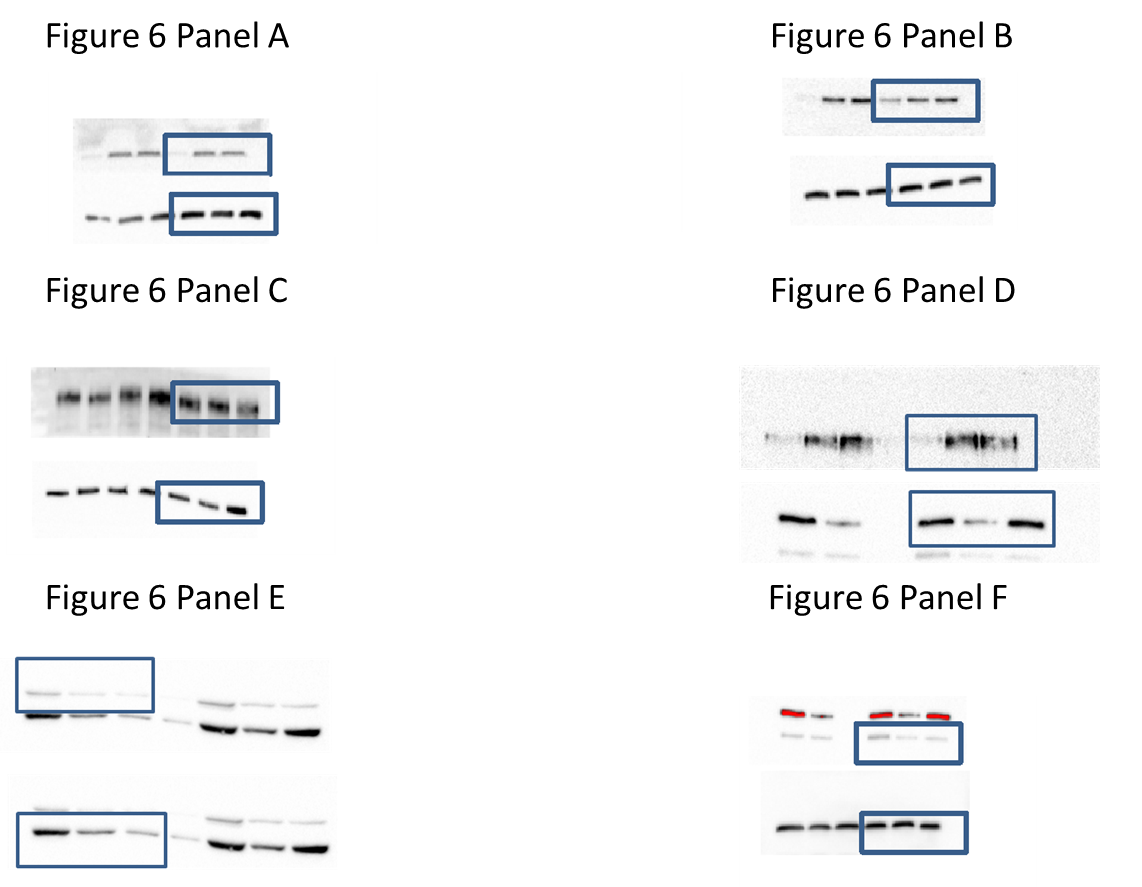

Supplement: Supplementary file 1 — Supplementary Information [file 41598_2019_53702_MOESM1_ESM.docx]
